# Prognostic Impact of Left Ventricular Ejection Fraction in Patients With Moderate Aortic Regurgitation: Potential Implications for Treatment Decision-Making

**DOI:** 10.3389/fcvm.2021.800961

**Published:** 2022-01-17

**Authors:** Qinghao Zhao, Bin Zhang, Yunqing Ye, Zhe Li, Qingrong Liu, Rui Zhao, Zhenyan Zhao, Weiwei Wang, Zikai Yu, Haitong Zhang, Zhenya Duan, Bincheng Wang, Junxing Lv, Shuai Guo, Yanyan Zhao, Runlin Gao, Haiyan Xu, Yongjian Wu

**Affiliations:** ^1^Department of Cardiology, Fuwai Hospital, National Center for Cardiovascular Diseases, Chinese Academy of Medical Sciences and Peking Union Medical College, Beijing, China; ^2^Department of Cardiovascular Surgery, Fuwai Hospital, National Center for Cardiovascular Diseases, Chinese Academy of Medical Sciences and Peking Union Medical College, Beijing, China; ^3^Medical Research & Biometrics Center, Fuwai Hospital, National Center for Cardiovascular Diseases, Chinese Academy of Medical Sciences and Peking Union Medical College, Beijing, China

**Keywords:** aortic regurgitation, left ventricular systolic dysfunction, mortality, heart failure, intervention

## Abstract

**Background:**

The prognostic impact and optimal treatment of left ventricular systolic dysfunction in patients with moderate aortic regurgitation (AR) remain unknown. We aimed to assess the prognostic value of left ventricular ejection fraction (LVEF) in patients with moderate AR and explore the potential benefits of aortic valve intervention (AVI).

**Methods:**

In total, 1,211 consecutive patients with moderate AR (jet width, 25–64% of LV outflow tract; vena contracta, 0.3–0.6 cm; regurgitant volume, 30–59 mL/beat; regurgitant fraction, 30–49%; effective regurgitation orifice, 0.10–0.29 cm^2^) prospectively registered between April and June 2018 at 46 academic hospitals were included. The primary outcome was a composite of death or hospitalization for heart failure (HHF). The optimal LVEF threshold for predicting the primary outcome was determined through the penalized spline shape and maximally selected rank statistics.

**Results:**

During the 2-year follow-up, 125 deaths or HHF occurred. In the penalized splines, the relative hazard of death or HHF monotonically increased with decreasing LVEF. In the multivariate analysis, LVEF ≤55% was identified as the best threshold for independently predicting death or HHF under medical treatment (adjusted hazard ratio [HR]: 2.18; 95% confidence interval [CI] 1.38–3.42; *P* = 0.001), with substantial incremental values (integrated discrimination improvement index = 0.018, *P* = 0.030; net reclassification improvement index = 0.225, *P* = 0.006; likelihood ratio test *P* < 0.001). Among patients with LVEF 35–55%, AVI within 6 months of diagnosis was associated with a reduced risk of death or HHF compared with medical treatment alone (adjusted HR: 0.15; 95% CI: 0.04–0.50; *P* = 0.002), whereas this benefit was markedly attenuated when LVEF was ≤35% (adjusted HR: 0.65; 95% CI: 0.21–1.97; *P* = 0.441, P-interactio*n* = 0.010) or >55% (adjusted HR: 0.40; 95% CI: 0.14–1.15; *P* = 0.089, P-interactio*n* = 0.723).

**Conclusions:**

LVEF is an independent and incremental prognostic factor in patients with moderate AR, with LVEF ≤55% being a robust marker of poor prognosis. Patients with LVEF 35–55% may benefit from early surgical correction of moderate AR. Further studies are warranted to validate our findings in a randomized setting.

**Registration:**

China Valvular Heart Disease Study (China-VHD study, NCT03484806); clinicaltrials.gov/ct2/show/NCT03484806.

## Introduction

The prevalence of aortic regurgitation (AR), a common valvular heart disease (VHD), increases sharply with age (1). Large population-based epidemiological studies reported that 2.0–2.3% of people aged >70 years have moderate or greater AR (1, 2). Additionally, left ventricular systolic dysfunction (LVSD) is highly prevalent in older adults, affecting up to 11% of the community-dwelling elderly population (3). Therefore, significant AR and LVSD often coexist in the elderly population, with a predominance of moderate AR (4). Whether as a direct cause or a comorbid condition of LVSD, AR imposes a significant preload and afterload burden on the left ventricle (LV), thereby exacerbating systolic dysfunction (5). In this case, the presence of moderate AR may not be as benign in patients with LVSD as in those with normal LV systolic function; however, relevant data are still lacking.

As a common indicator of LVSD, reduced LV ejection fraction (LVEF) <50 or 55% is known to be a potent predictor of poor prognosis and serves as a reasonable indication for aortic valve intervention (AVI) in patients with severe AR (6, 7). However, the prognostic value of LVEF and its best cutoff for risk prediction in patients with moderate AR remain unclear. Moreover, among patients with the failing LV, reduced hemodynamic burden following mechanical relief from significant aortic valve disease may substantially improve long-term prognosis (8). Recent studies have reported that patients with moderate aortic stenosis and LVSD could derive considerable prognostic improvements from AVI (9, 10), but no data is available regarding the potential benefits of AVI in patients with moderate AR and LVSD. In this case, recommendations for moderate AR are based on expert opinion, with AVI being indicated only when undergoing other cardiac or aortic surgeries (Class IIa, Level C) (7).

Thus, this study aimed to assess the prognostic impact of reduced LVEF on patients with moderate AR, and determine the optimal LVEF cutoff for predicting poor prognosis under conservative treatment, and explore the potential effectiveness of AVI in improving symptoms and outcomes of patients with moderate AR and LVSD.

## Methods

### Study Population

The China Valvular Heart Disease Study (China-VHD, NCT03484806) is a nationwide, multicenter, prospective cohort study involving adult patients (≥18 years) with VHD. Patients were recruited between April and June 2018 from inpatient wards and outpatient clinics of 46 large academic hospitals throughout China (Supplementary Methods). In this study, patients with moderate or severe native VHD, as defined by echocardiography using an integrative approach according to the 2014 American Heart Association/American College of Cardiology (AHA/ACC) guidelines (11), endocarditis, and previous valvular intervention were consecutively enrolled. Institutional Review Boards at the National Center for Cardiovascular Diseases of China approved the study protocol. Written informed consent was obtained from all eligible participants.

In total, 13,917 consecutive adult patients with VHD were enrolled. Moderate AR was observed in 2,365 patients (jet width, 25–64% of LV outflow tract; vena contracta, 0.3–0.6 cm; regurgitant volume, 30–59 mL/beat; regurgitant fraction, 30–49%; effective regurgitation orifice, 0.10–0.29 cm^2^) (11). We excluded patients with previous AVI, acute AR resulting from aortic dissection or active endocarditis, ≥moderate aortic or mitral stenosis, ≥moderate primary or severe secondary mitral regurgitation (MR), dilated and hypertrophic cardiomyopathy, congenital heart disease (except dysmorphic aortic valve), acute aortic syndrome, aortic rupture, and acute myocardial infarction (MI) within 90 days. Since secondary MR is a common comorbid condition in patients with LVSD presenting with LV dilatation, patients with moderate secondary MR were not excluded to avoid selection bias in LV dimensions. Finally, 1,211 patients were included.

### Echocardiography

All patients underwent comprehensive transthoracic 2-dimensional and Doppler echocardiography using standard ultrasound systems. Chamber quantification was performed according to the echocardiography guidelines (12). LVEF was estimated using the biplane-modified Simpson method. Aortic measurements were obtained from the parasternal long-axis window. Pulmonary artery pressure was calculated from the maximum peak tricuspid regurgitation velocity using the Bernoulli equation. Quality control of the echocardiographic measurements is detailed in Supplementary Methods.

### Clinical Outcomes

The primary outcome was a composite of all-cause death or hospitalization for heart failure (HHF). HHF was defined as hospitalization with a primary diagnosis of heart failure (HF) where the patient exhibited new or worsening symptoms and evidence of HF and received initiation or intensification of treatment specifically for HF. The composite of all-cause death or HHF under medical treatment (MT) was also assessed in the whole cohort with censoring at the time of AVI if performed. Hence, in patients who underwent AVI, the time between baseline echocardiography and AVI was considered as medically managed follow-up. Outcome data were obtained from patient visits, medical records, and telephone interviews (detailed in Supplementary Methods).

### Statistical Analyses

Continuous data are summarized as mean ± standard deviation or median with interquartile range and compared using the Student *t*-test or Mann-Whitney *U*-test, as appropriate. Categorical data are presented as percentages and compared using the chi-square test.

To assess the prognostic impact of LVEF on moderate AR, we initially used penalized splines (P-splines) to depict the shape of the association between LVEF and the primary outcome in overall, medically, and AVI managed patients, where AVI was treated in a time-dependent manner in which the time between baseline echocardiography and AVI was considered as medically managed follow-up. In conjunction with the P-spline shape under MT, we employed the maximally selected rank statistics method to determine the most significant LVEF cutoff for predicting death or HHF under MT, with the largest standardized log-rank statistics over all possible cutoff points (13). Based on the selected LVEF threshold, we estimated the cumulative incidences of outcomes under MT using the Kaplan-Meier method and assessed hazard ratios (HRs) with 95% confidence intervals (CIs) using Cox proportional-hazards models. In the multivariate analysis, the least absolute shrinkage and selection operator (LASSO)-penalized Cox regression was used to identify the variables associated with death or HHF under MT with additional regard for clinical relevance (detailed in Supplementary Methods) (14). The following variables were selected in the final adjusted model: age, body mass index (BMI), atrial fibrillation (AF), prior MI, prior coronary artery bypass grafting (CABG), chronic kidney disease, New York Heart Association (NYHA) class III/IV, hemoglobin, LV end-systolic diameter (LVESD) >50 mm, pulmonary hypertension, and EuroSCORE-II. In addition, we also assessed the association pattern of LVEF with the primary outcome and the best threshold for risk prediction in an age- and sex-matched population without left-sided VHD for comparison (detailed in Supplementary Methods).

To analyze the incremental value of LVEF, we introduced LVEF as a continuous or categorical variable (dichotomized by the selected LVEF cutoff) to a base model (including all covariables in the abovementioned multivariate analysis) and compared the model performance. The C-index, integrated discrimination improvement (IDI) index, and net reclassification improvement (NRI) index were calculated to assess the discrimination properties. The likelihood ratio (LR) test and Bayesian information criteria were used to evaluate the calibration properties. Decision curve analysis was also performed to further validate the incremental value of LVEF.

To investigate the effectiveness of AVI in prognostic improvement among patients with moderate AR and LVSD, we delineated the pattern of the relative risk for the primary outcome after AVI vs. under MT according to LVEF using the P-spline. Based on the P-spline shape and the best LVEF threshold for risk prediction, we divided the LVEF into three ranges (≤35%, 35–55%, >55%) and assessed the impact of AVI on the primary outcome of each using inverse probability of treatment weighted (IPTW) Cox regression models (detailed in Supplementary Methods). The following variables were used to construct the IPTW weights: age, sex, BMI, systolic blood pressure, AF, coronary artery disease (CAD), prior MI, prior CABG, aortic disease, chronic kidney disease, NYHA class III/IV, hemoglobin, LVEF, LVESD >50 mm, secondary MR, pulmonary hypertension, EuroSCORE-II, use of angiotensin-converting enzyme inhibitors/angiotensin receptor blockers, and use of beta-blockers. Of note, to avoid immortal-time bias (15), the time-zero of the Cox models was the time of AVI for the recipients and day 15 following the baseline echocardiography for the non-recipients. Moreover, given the changing nature of LVEF and AR severity during follow-up, we assessed only the prognostic impact of early AVI treatment, defined as AVI within 6 months of the baseline echocardiography, excluding patients who underwent AVI after 6 months. Among the same population, we employed river plots to demonstrate the 2-year temporal changes in symptom status (NYHA functional classification) under MT and early AVI treatment (within 6 months). Statistical significance was set at two-tail *P* < 0.05. All analyses were performed using R 4.0.2 (R Foundation for Statistical Computing, Vienna, Austria).

## Results

### Baseline Characteristics and Treatment

The baseline characteristics are presented in Table 1. The median age of the 1,211 patients was 66 (57–73) years; 67.6% were male. Hypertension (59.4%), diabetes (12.6%), AF (18.2%), CAD (41.3%), and aortic disease (14.7%) were highly prevalent. Among them, 24.1% had NYHA class III/IV HF. The median LVEF was 59% (52–64%) and the median EuroSCORE-II was 1.0 (0.7–1.9).

**Table 1:** Baseline characteristics.

**Characteristic**	**Whole cohort** **(***n*** = 1,211)**	**LVEF ≤55%** **(***n*** = 468)**	**LVEF >55%** **(***n*** = 743)**	***P*****-value** **(≤55 vs. >55%)**
**Demographics and vital signs**				
Age, yr [Median (IQR)]	66 (57–73)	67 (59–75)	65 (56–72)	0.009
Male, no. (%)	819 (67.6%)	350 (74.8%)	469 (63.1%)	<0.001
BMI, kg/m^2^ [Median (IQR)]	23.8 (21.5–26.1)	23.7 (21.2–25.9)	23.9 (21.7–26.2)	0.098
BSA, m^2^ (Mean ± SD)	1.83 ± 0.19	1.82 ± 0.19	1.83 ± 0.18	0.379
Heart rate, bpm [Median (IQR)]	72 (65–81)	75 (66–85)	71 (64–80)	<0.001
SBP, mmHg [Median (IQR)]	130 (120–144)	130 (119–144)	130 (120–145)	0.368
DBP, mmHg [Median (IQR)]	73 (65–80)	71 (63–80)	74 (66–80)	0.049
**Risk factors and comorbidities**				
Current Smoker, no. (%)	234 (19.3%)	107 (22.9%)	127 (17.1%)	0.014
Hypertension, no. (%)	719 (59.4%)	271 (57.9%)	448 (60.3%)	0.410
Diabetes, no. (%)	153 (12.6%)	63 (13.5%)	90 (12.1%)	0.493
Dyslipidemia, no. (%)	196 (16.2%)	52 (11.1%)	144 (19.4%)	<0.001
Atrial fibrillation, no. (%)	221 (18.2%)	110 (23.5%)	111 (14.9%)	<0.001
Coronary artery disease, no. (%)*	500 (41.3%)	210 (44.9%)	290 (39%)	0.045
Prior Myocardial infarction, no. (%)	78 (6.4%)	52 (11.1%)	26 (3.5%)	<0.001
Prior PCI, no. (%)	166 (13.7%)	75 (16.0%)	91 (12.2%)	0.065
Prior CABG, no. (%)	19 (1.6%)	14 (3.0%)	5 (0.7%)	0.003
Aortic disease, no. (%)^†^	178 (14.7%)	58 (12.4%)	120 (16.2%)	0.070
Cerebrovascular disease, no. (%)	134 (11.1%)	52 (11.1%)	82 (11.0%)	0.968
Peripheral artery disease, no. (%)	53 (4.4%)	12 (2.6%)	41 (5.5%)	0.011
Chronic obstructive pulmonary disease, no. (%)	74 (6.1%)	40 (8.5%)	34 (4.6%)	0.006
Chronic kidney disease, no. (%)	72 (5.9%)	42 (9.0%)	30 (4.0%)	0.001
**Baseline symptoms**				
Dyspnea, no. (%)	619 (51.1%)	297 (63.5%)	322 (43.3%)	<0.001
Chest pain, no. (%)	332 (27.4%)	119 (25.4%)	213 (28.7%)	0.217
NYHA functional classification, no. (%)				<0.001
I	560 (46.2%)	148 (31.6%)	412 (55.5%)	
II	359 (29.6%)	123 (26.3%)	236 (31.8%)	
III	217 (17.9%)	141 (30.1%)	76 (10.2%)	
IV	75 (6.2%)	56 (12%)	19 (2.6%)	
**Laboratory**				
Hemoglobin, g/L [Median (IQR)]	134 (121–146)	133 (121–146)	135 (122–147)	0.293
eGFR, ml/min/1.73 m^2^ (Mean ± SD)	85.3 ± 26.8	79.8 ± 25.5	88.9 ± 27.0	<0.001
LDL, mmol/L [Median (IQR)]	2.3 (1.8–2.9)	2.3 (1.8–2.8)	2.4 (1.9–3.0)	0.007
Total cholesterol, mmol/L [Median (IQR)]	4.0 (3.3–4.7)	3.9 (3.3–4.5)	4.0 (3.4–4.8)	0.002
NT–proBNP (*n* = 595), pg/ml [Median (IQR)]	539 (150–2105)	1652 (444–4527)	276 (98–733)	<0.001
BNP (*n* = 177), pg/ml [Median (IQR)]	49 (19–160)	156 (52–321)	28 (9–69)	<0.001
**Echocardiography**				
LVEF, % [Median (IQR)]	59 (52–64)	45 (35–52)	62 (60–65)	<0.001
LVESD, mm [Median (IQR)]	36 (31–42)	44 (38–52)	33 (30–36)	<0.001
LVESD >50 mm [Median (IQR)]	142 (11.7%)	139 (29.7%)	3 (0.4%)	<0.001
LVESDi, mm/m^2^ [Median (IQR)]^‡^	19.5 (17.2–23.5)	24.5 (20.6–28.7)	17.9 (16.2–20.0)	<0.001
LVEDD, mm [Median (IQR)]	54 (49–60)	60 (53–67)	51 (47–56)	<0.001
LVEDD >70 mm [Median (IQR)]	78 (6.4%)	74 (15.8%)	4 (0.5%)	<0.001
LVEDDi, mm/m^2^ [Median (IQR)]^‡^	29.8 (26.9–33.4)	33.1 (29.6–36.9)	28.4 (25.9–31.0)	<0.001
LAi, mm/m^2^ [Median (IQR)]^‡^	21.7 (19.3–25.0)	23.5 (20.9–27.5)	20.7 (18.8–23.6)	<0.001
Aortic valve morphology, no. (%)				0.183
Tricuspid	1124 (92.8%)	441 (94.2%)	683 (91.9%)	
Bicuspid	75 (6.2%)	25 (5.3%)	50 (6.7%)	
Unicuspid/Quadricuspid	12 (1.0%)	2 (0.4%)	10 (1.3%)	
Moderate secondary MR, no. (%)	159 (13.1%)	111 (23.7%)	48 (6.5%)	<0.001
≥moderate TR, no. (%)	191 (15.8%)	94 (20.1%)	97 (13.1%)	0.001
Pulmonary hypertension, no. (%)	236 (19.5%)	125 (26.7%)	111 (14.9%)	<0.001
Ascending aortic diameter, mm	36 (31–41)	36 (32–41)	36 (31–41)	0.173
>45 mm, no. (%)	121 (10.1%)	40 (8.5%)	81 (10.9%)	0.201
**Reasons for admission**				0.767
Admitted for VHD^§^	398 (32.9%)	150 (32.1%)	248 (33.4%)	
Admitted for cardiovascular diseases other than VHD	712 (58.8%)	281 (60.0%)	431 (58.0%)	
Admitted for non–cardiovascular diseases	101 (8.3%)	37 (7.9%)	64 (8.6%)	
**Etiology^||^**				<0.001
Degenerative	442 (36.5%)	143 (30.6%)	299 (40.2%)	
Secondary	473 (39.1%)	222 (47.4%)	251 (33.8%)	
Rheumatic	78 (6.4%)	24 (5.1%)	54 (7.3%)	
Congenital	119 (9.8%)	36 (7.7%)	83 (11.2%)	
Autoimmune	5 (0.4%)	3 (0.6%)	2 (0.3%)	
**EuroSCORE–II [Median (IQR)]**	1.0 (0.7–1.9)	1.4 (0.9–2.5)	0.9 (0.6–1.4)	<0.001
**Intervention**	306 (25.3%)	116 (24.8%)	190 (25.6%)	0.759
Aortic valve repair, no. (%)	15 (1.2%)	6 (1.3%)	9 (1.2%)	0.914
SAVR, no. (%)	283 (23.4%)	106 (22.6%)	177 (23.8%)	0.638
TAVR, no. (%)	8 (0.7%)	4 (0.9%)	4 (0.5%)	0.514
Concomitant cardiac or aortic surgery	170 (14.0%)	60 (12.8%)	110 (14.8%)	0.331
CABG, no. (%)	53 (4.4%)	19 (4.1%)	34 (4.6%)	0.668
Aortic surgery, no. (%)	73 (6.0%)	17 (3.6%)	56 (7.5%)	0.004
Other cardiac surgery, no. (%) #	81 (6.7%)	35 (7.5%)	46 (6.2%)	0.385
**Medication use**				
Beta–blocker, no. (%)	777 (64.2%)	327 (69.9%)	450 (60.6%)	0.001
ACEI/ARB, no. (%)	631 (52.1%)	276 (59%)	355 (47.8%)	<0.001
ARNI, no. (%)	23 (1.9%)	20 (4.3%)	3 (0.4%)	<0.001
Diuretics, no. (%)	744 (61.4%)	367 (78.4%)	377 (50.7%)	<0.001
Digitalis, no. (%)	293 (24.2%)	159 (34%)	134 (18%)	<0.001
Warfarin, no. (%)	455 (37.6%)	176 (37.6%)	279 (37.6%)	0.984
New oral anticoagulants, no. (%)	82 (6.8%)	40 (8.5%)	42 (5.7%)	0.054
Antiplatelet agents, no. (%)	666 (55.0%)	270 (57.7%)	396 (53.3%)	0.134

*ACEI, angiotensin-converting enzyme inhibitors; ARB, angiotensin receptor blocker; ARNI, angiotensin receptor-neprilysin inhibitor; BMI, body mass index; BNP, B-type natriuretic peptide; BSA, body surface area; CABG, coronary artery bypass grafting; DBP, diastolic blood pressure; eGFR, estimated glomerular filtration rate; EuroSCORE-II, European System for Cardiac Operative Risk Evaluation; IQR, interquartile range; LA, left atrium end-diastolic dimension; LDL, low-density lipoproteins; LVEDD, left ventricular end-diastolic dimension; LVEF, left ventricular ejection fraction; LVESD, left ventricular end-systolic dimension; MR, mitral regurgitation; NT-proBNP, N-terminal pro-B-type natriuretic peptide; NYHA, New York Heart Association; PCI, percutaneous coronary intervention; SBP, systolic blood pressure; SAVR, surgical aortic valve replacement; TAVR, transcatheter aortic valve replacement; TR, tricuspid regurgitation; VHD, valvular heart disease*.

* *Including CAD, previous myocardial infarction, and history of PCI and CABG procedures. Myocardial infarction within 90 days has been excluded from the study population*.

† *Including aortic aneurysms, atherosclerotic and inflammatory aortic disease, genetic diseases (e.g., Marfan syndrome), and congenital abnormalities. Acute aortic syndromes (including aortic dissection, intramural hematoma, and penetrating atherosclerotic ulcer) and aortic rupture have been excluded from the study population*.

‡ *Dimensions of left ventricle and left atrium indexed to body surface area*.

§ *indicating hospitalization for the diagnosis and treatment of valvular heart disease*.

|| *The etiologies of AR were defined based on echocardiographic findings in conjunction with clinical profiles and surgical findings (if available)*.

# *Including heart valve surgery other than aortic valve intervention, antiarrhythmic surgery and other open-heart surgery*.

Within 2 years, 306 (25.3%) patients underwent AVI, 298 of them within 6 months of the baseline echocardiography. Most patients underwent surgical aortic valve replacement (SAVR) (283/306 [92.4%]), while 4.9% (15/306) and 2.6% (8/306) were treated with aortic valve repair and transcatheter aortic valve replacement (TAVR), respectively. About half of the patients who underwent AVI also had concomitant cardiac or aortic surgery (157/306 [51.3%]), with CABG (52/157 [33.1%]) and aortic surgery (69/157 [43.9%]) being the most common (Table 2).

**Table 2:** Baseline characteristics according to treatment strategies.

**Characteristic**	**Aortic valve intervention** **(***n*** = 306)**	**Medical treatment** **(***n*** = 905)**	***P*** **-value**
**Demographics and vital signs**			
Age, yr [Median (IQR)]	58 (50–66)	68 (61–75)	<0.001
Male, no. (%)	238 (77.8%)	581 (64.2%)	<0.001
BMI, kg/m^2^ [Median (IQR)]	24.2 (22.1–26.6)	23.7 (21.3–26.0)	0.018
BSA, m^2^ (Mean ± SD)	1.88 ± 0.17	1.81 ± 0.19	<0.001
Heart rate, bpm [Median (IQR)]	75 (65.75–80)	72 (64–81)	0.122
SBP, mmHg [Median (IQR)]	130 (120–141)	130 (120–145)	0.056
DBP, mmHg [Median (IQR)]	70 (62–78)	74 (66–80)	<0.001
**Risk factors and comorbidities**			
Current smoker, no. (%)	65 (21.2%)	169 (18.7%)	0.329
Hypertension, no. (%)	160 (52.3%)	559 (61.8%)	0.004
Diabetes, no. (%)	9 (2.9%)	144 (15.9%)	<0.001
Dyslipidemia, no. (%)	52 (17.0%)	144 (15.9%)	0.658
Atrial fibrillation, no. (%)	24 (7.8%)	197 (21.8%)	<0.001
Coronary artery disease, no. (%)	72 (23.5%)	428 (47.3%)	<0.001
Prior myocardial infarction, no. (%)	12 (3.9%)	66 (7.3%)	0.029
Prior PCI, no. (%)	15 (4.9%)	151 (16.7%)	<0.001
Prior CABG, no. (%)	2 (0.7%)	17 (1.9%)	0.185
Aortic disease, no. (%)	58 (19.0%)	120 (13.3%)	0.017
Cerebrovascular disease, no. (%)	14 (4.6%)	120 (13.3%)	<0.001
Peripheral artery disease, no. (%)	3 (1.0%)	50 (5.5%)	<0.001
Chronic obstructive pulmonary disease, no. (%)	10 (3.3%)	64 (7.1%)	0.011
Chronic kidney disease, no. (%)	3 (1.0%)	69 (7.6%)	<0.001
**Baseline symptoms**			
Dyspnea, no. (%)	213 (69.6%)	406 (44.9%)	<0.001
Chest pain, no. (%)	75 (24.5%)	257 (28.4%)	0.184
NYHA functional classification, no. (%)			<0.001
I	84 (27.5%)	476 (52.6%)	
II	132 (43.1%)	227 (25.1%)	
III	76 (24.8%)	141 (15.6%)	
IV	14 (4.6%)	61 (6.7%)	
**Laboratory**			
Hemoglobin, g/L [Median (IQR)]	141 (129–151)	132 (119–144)	<0.001
eGFR, ml/min/1.73m^2^ (Mean ± SD)	92.3 ± 25.4	82.7 ± 26.8	<0.001
LDL, mmol/L [Median (IQR)]	2.4 (1.9–2.9)	2.3 (1.8–2.9)	0.440
Total cholesterol, mmol/L [Median (IQR)]	4.0 (3.3–4.7)	4.0 (3.3–4.7)	0.908
NT–proBNP (*n* = 595), pg/ml [Median (IQR)]	278 (83–1111)	748 (217–2698)	<0.001
BNP (*n* = 177), pg/ml [Median (IQR)]	121 (36–170)	42 (18–157)	0.246
**Echocardiography**			
LVEF, % [Median (IQR)]	60 (53–64)	59 (51–64)	0.930
LVESD, mm [Median (IQR)]	38 (33–44)	35 (31–42)	<0.001
LVESD >50 mm [Median (IQR)]	39 (12.7%)	103 (11.4%)	0.525
LVESDi, mm/m^2^ [Median (IQR)]	20.4 (17.9–24.0)	19.3 (17.0–23.1)	0.005
LVEDD, mm [Median (IQR)]	58 (53–64)	53 (48–59)	<0.001
LVEDD >70 mm [Median (IQR)]	25 (8.2%)	53 (5.9%)	0.164
LVEDDi, mm/m^2^ [Median (IQR)]	31.1 (27.9–34.1)	29.3 (26.4–33.1)	<0.001
LAi, mm/m^2^ [Median (IQR)]	20.4 (18.1–23.4)	22.1 (19.7–25.5)	<0.001
Aortic valve morphology, no. (%)			<0.001
Tricuspid	253 (82.7%)	871 (96.2%)	
Bicuspid	44 (14.4%)	31 (3.4%)	
Unicuspid/Quadricuspid	9 (2.9%)	3 (0.3%)	
Moderate secondary MR, no. (%)	18 (5.9%)	141 (15.6%)	<0.001
≥moderate TR, no. (%)	15 (8.2%)	176 (5.9%)	<0.001
Pulmonary hypertension, no. (%)	38 (12.4%)	198 (21.9%)	<0.001
Ascending aortic diameter, mm	39 (35–45)	35 (30–40)	<0.001
>45 mm, no. (%)	68 (22.2%)	53 (5.9%)	<0.001
**Reasons for admission**			<0.001
Admitted for VHD	238 (77.8%)	160 (17.7%)	
Admitted for cardiovascular diseases other than VHD	57 (18.6%)	655 (72.4%)	
Admitted for non–cardiovascular diseases	11 (3.6%)	90 (9.9%)	
**Etiology**			<0.001
Degenerative	96 (31.4%)	346 (38.2%)	
Secondary	86 (28.1%)	387 (42.8%)	
Rheumatic	26 (8.5%)	52 (5.7%)	
Congenital	72 (23.5%)	47 (5.2%)	
Autoimmune	1 (0.3%)	4 (0.4%)	
**EuroSCORE–II [Median (IQR)]**	0.9 (0.6–1.2)	1.1 (0.8–2.1)	<0.001
**Intervention**			
Aortic valve repair, no. (%)	15 (4.9%)	–	–
SAVR, no. (%)	283 (92.4%)	–	–
TAVR, no. (%)	8 (2.6%)	–	–
Concomitant cardiac or aortic surgery	157 (51.3%)		
CABG, no. (%)	52 (17.0%)	–	–
Aortic surgery, no. (%)	69 (22.5%)	–	–
Other cardiac surgery, no. (%)^||^	71 (23.2%)	–	–
Isolated cardiac or aortic surgery without AVI		13 (1.4%)	
CABG, no. (%)	–	6 (0.7%)	–
Aortic surgery, no. (%)	–	4 (0.4%)	–
Other cardiac surgery, no. (%)	–	10 (1.1%)	–
**Medication use**			
Beta–blocker, no. (%)	205 (67.0%)	572 (63.2%)	0.230
ACEI/ARB, no. (%)	136 (44.4%)	495 (54.7%)	0.002
ARNI, no. (%)	2 (0.6%)	21 (2.3%)	0.087
Diuretics, no. (%)	282 (92.2%)	462 (51.0%)	<0.001
Digitalis, no. (%)	146 (47.7%)	147 (16.2%)	<0.001
Warfarin, no. (%)	293 (95.8%)	162 (17.9%)	<0.001
New oral anticoagulants, no. (%)	4 (1.3%)	78 (8.6%)	<0.001
Antiplatelet agents, no. (%)	93 (30.4%)	573 (63.3%)	<0.001

*eGFR, estimated glomerular filtration rate; LDL, low-density lipoproteins*.

### Association Between LVEF and Clinical Outcomes

**Figure 1:**
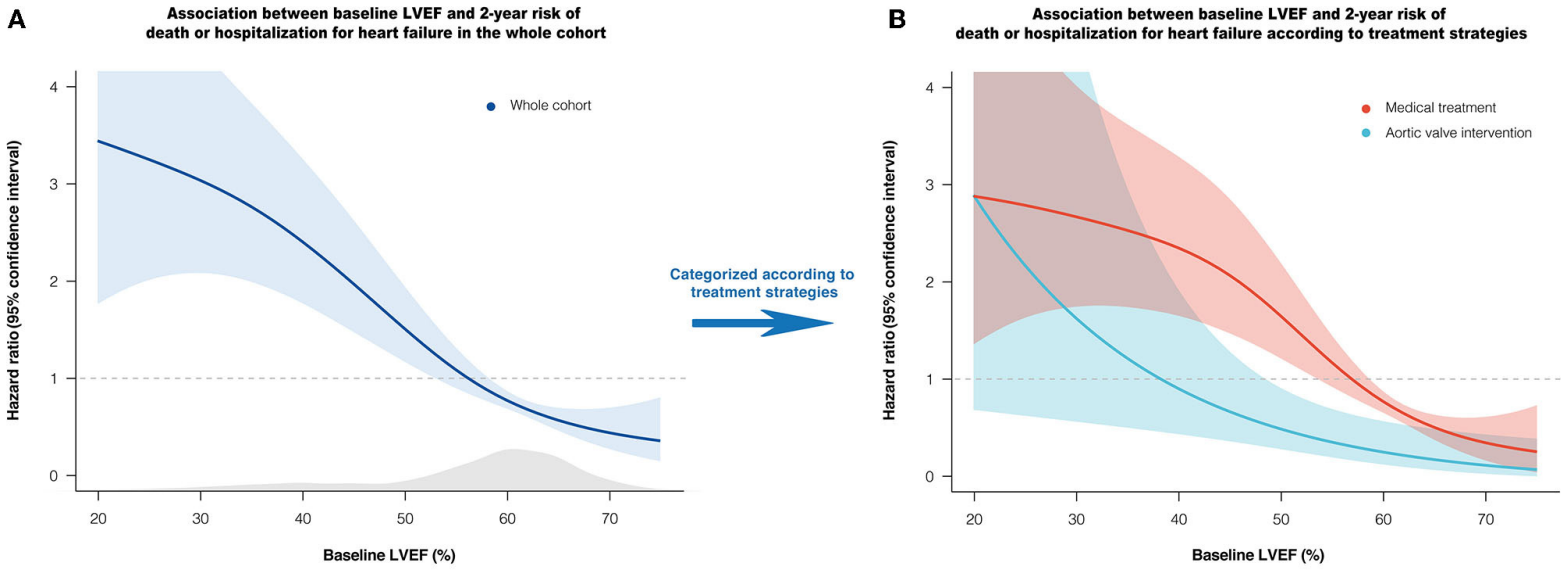
Association between baseline LVEF and relative hazard of 2-year death or HHF. Penalized spline curves demonstrate the shape of the association in overall **(A)**, medically and AVI managed patients **(B)**, with 95% confidence interval. The gray area underneath the curve indicates the density of the population. The horizontal line at HR = 1 represents the mean risk of the cohort. AVI, aortic valve intervention; HHF, hospitalization for heart failure; LVEF, left ventricular ejection fraction.

During the median follow-up of 24.4 (23.4–24.9) months, death or HHF occurred in 125 (10.3%) patients (60 deaths, 75 HHF). In P-splines, the relative hazards of death or HHF in overall, medically, and AVI managed patients all presented a monotonic increase with decreasing LVEF (Figure 1). Under MT, the risk began to exceed the mean risk of the entire cohort when LVEF declined to ~55–60%, with a remarkably sharp increase with a continued LVEF decline. AVI considerably alleviated the risk of death or HHF, with the P-spline curve consistently beneath that of MT across the LVEF (Figure 1B). LVEF was the most contributive independent predictor of death or HHF under MT (per 10% increase: adjusted HR: 0.65; 95% CI: 0.53–0.79; *P* = 0.008) or AVI treatment (per 10% increase: adjusted HR: 0.65; 95% CI: 0.43–0.97; *P* = 0.036) by LASSO-penalized Cox regression (Supplementary Figures 3–6).

**Figure 2:**
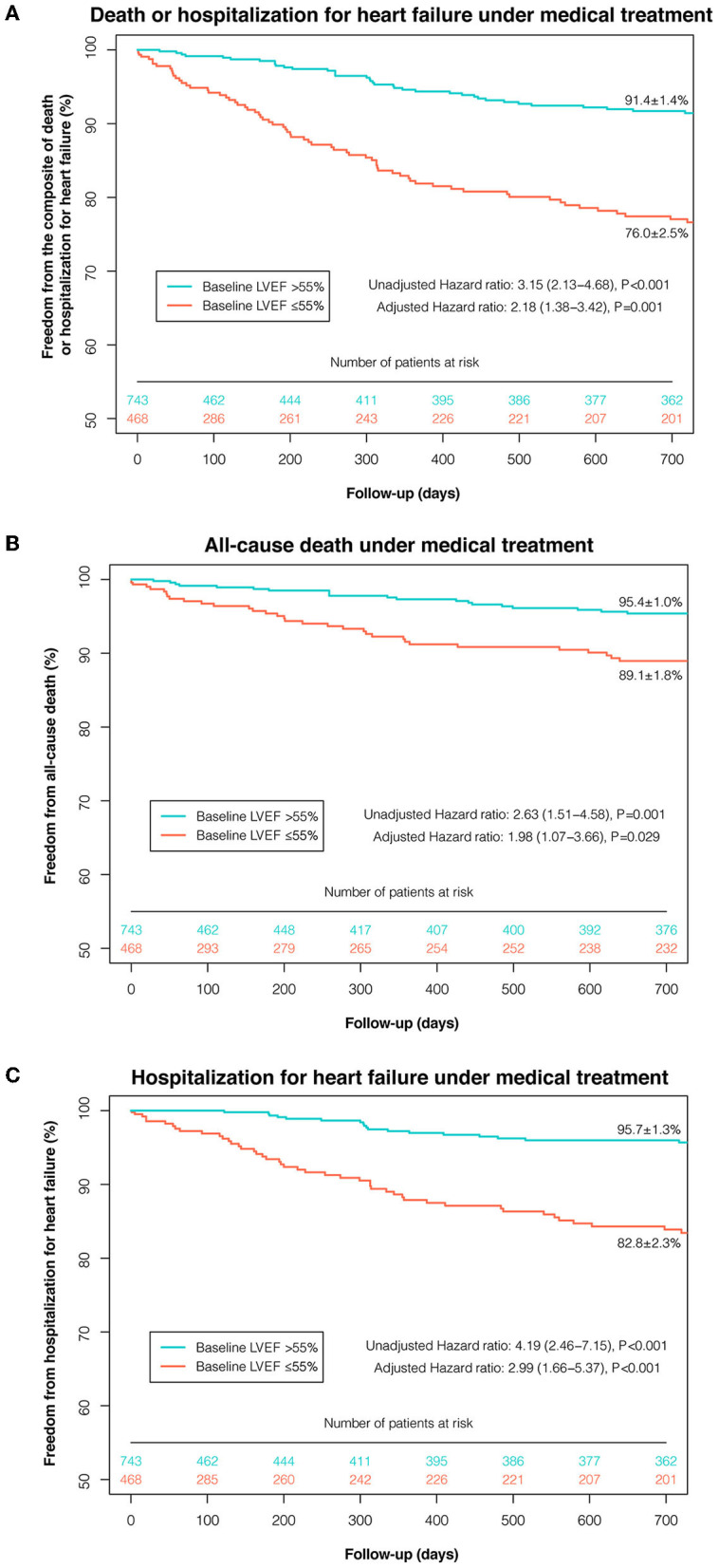
Kaplan-Meier curves of event-free survival under medical treatment according to the selected LVEF threshold. Kaplan-Meier curves of freedom from the composite of death or HHF **(A)**, death **(B)**, and HHF **(C)** under medical treatment were plotted according to the selected LVEF threshold (≤55 and >55%). AVI, aortic valve intervention; HHF, hospitalization for heart failure; LVEF, left ventricular ejection fraction.

Based on the maximally selected rank statistics and the P-spline shape, LVEF ≤55% was identified as the most significant threshold for predicting 2-year death or HHF in patients with moderate AR under MT (Supplementary Figure 7), which was higher than the threshold for the age- and sex-matched population without left-sided VHD (LVEF ≤48%, Supplementary Figures 9, 10). After multivariate adjustment, LVEF ≤55% was independently associated with a higher risk of death or HHF under MT (adjusted HR: 2.18; 95% CI: 1.38–3.42; *P* = 0.001) (Figure 2). The association between LVEF ≤55% and the risk of death or HHF remained consistent in the subgroup analyses (stratified by age, sex, symptoms, CAD, secondary MR, AR etiology, and EuroSCORE-II), with no significant interactions (all P-interaction >0.05) (Supplementary Table 5).

### Incremental Prognostic Value of LVEF in Moderate AR

The addition of LVEF to the base model substantially improved the predictive power, either as a continuous variable (IDI=0.019, *P* = 0.032; NRI=0.219, *P* = 0.012; LR test *P* < 0.001) or as a categorical variable dichotomized by 55% (IDI=0.018, *P* = 0.030; NRI=0.225, *P* = 0.006; LR test *P* < 0.001) (Table 3). The superiority of introducing LVEF to the model persisted in the decision curve analysis, with a higher net benefit (Supplementary Figure 8).

**Table 3:** Comparison of risk–prediction models with and without baseline LVEF for the composite of death or HHF under medical treatment.

	Base model*	Base model*+ LVEF (continuous)	Base model*+ LVEF (dichotomized by 55%)
Discrimination			
C–statistic	0.76	0.78	0.78
IDI (95% CI)	Reference	0.019 (0.001–0.051) *P* = 0.032	0.018 (0.001–0.044) *P* = 0.030
NRI (95% CI)	Reference	0.219 (0.066–0.343) *P* = 0.012	0.225 (0.104–0.333) *P* = 0.006
Calibration			
LR test	Reference	*P* <0.001	*P* <0.001
BIC	1,366	1,358	1,359

*BIC, Bayesian information criteria; CI, confidence interval; HHF, hospitalization for heart failure; IDI, integrated discrimination improvement; LR, likelihood ratio; LVEF, left ventricular ejection fraction; NRI, net reclassification improvement*.

* *Base model adjusted for age, body mass index, atrial fibrillation, prior myocardial infarction, prior coronary artery bypass grafting, chronic kidney disease, New York Heart Association class III/IV, hemoglobin, left ventricular end–systolic diameter >50 mm, pulmonary hypertension, and EuroSCORE–II*.

### Impact of Treatment Strategies on Clinical Outcomes According to LVEF

**Figure 3:**
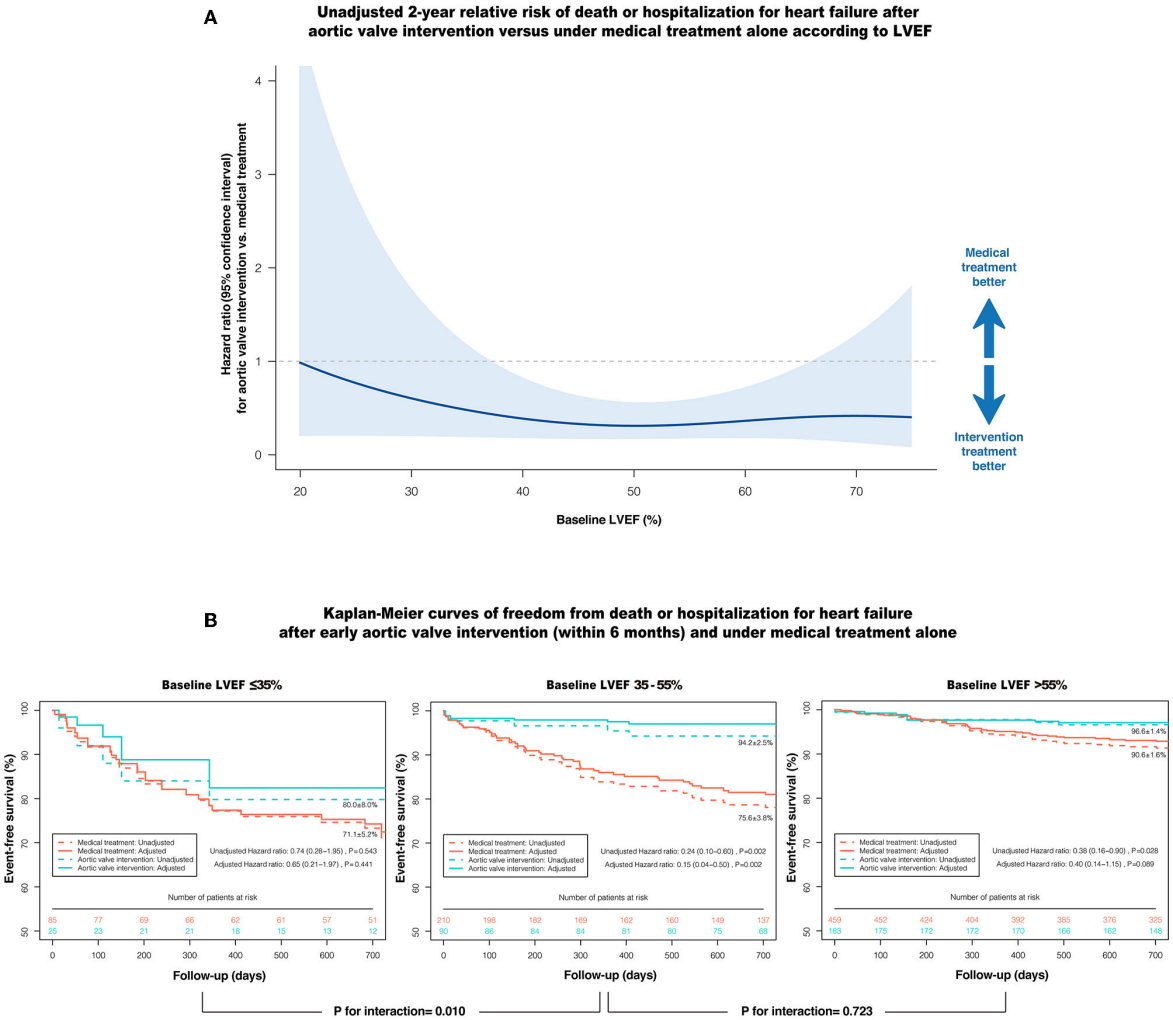
Impact of AVI on the composite of death or HHF according to baseline LVEF. **(A)** The unadjusted relative risk of death or HHF after AVI vs. under medical treatment alone according to LVEF, with 95% confidence interval. The shaded area entirely below the horizontal line (HR = 1) denotes the upper bound of the 95% confidence interval is <1, indicating AVI is prognostically more favorable at this LVEF. **(B)** Kaplan-Meier curves of event-free survival after early AVI (within 6 months) and under medical treatment alone according to the LVEF ranges, with (solid line) and without (dashed line) inverse probability treatment weighting adjustment. AVI, aortic valve intervention; HHF, hospitalization for heart failure; LVEF, left ventricular ejection fraction.

Figure 3A shows the unadjusted relative risk of death or HHF after AVI vs. under MT according to LVEF. Among patients with LVSD (LVEF ≤55%), AVI showed a protective effect against death or HHF compared with MT alone at an LVEF of about 35–55%; however, this effect began to lose statistical significance when LVEF decreased to ~35%.

After adjusting for the clinically relevant factors and anti-HF medications using IPTW, early AVI (within 6 months) was strongly associated with a reduced risk of death or HHF in patients with LVEF 35–55% compared with MT (adjusted HR: 0.15; 95% CI: 0.04–0.50; *P* = 0.002), whereas this prognostic benefit was markedly attenuated when LVEF was ≤35% (adjusted HR: 0.65; 95% CI: 0.21–1.97; *P* = 0.441), with a significant interaction (P-interactio*n* = 0.010) (Figure 3B). In contrast, patients with LVEF >55% had a low 2-year cumulative incidence of death or HHF, even under MT alone (AVI: 96.6 ± 1.4% vs. MT: 90.6 ± 1.6%), with no significant difference in the risk of death or HHF after multivariate adjustment (adjusted HR: 0.40; 95% CI: 0.14–1.15; *P* = 0.089). In terms of the individual components of the composite outcome, early AVI was also significantly associated with a lower risk of death (adjusted HR: 0.05; 95% CI: 0.01–0.44; *P* = 0.006) and HHF (adjusted HR: 0.18; 95% CI: 0.05–0.66; *P* = 0.010) vs. MT at an LVEF of 35–55%. However, the advantages of early AVI in mortality and HHF were not obvious when LVEF decreased to ≤35% or increased to >55% (all *P* > 0.05) (Table 4). Moreover, the results of the sensitivity analyses in patients without CAD, aortic disease, secondary MR and concomitant cardiac or aortic surgery were consistent with the overall findings (Table 4).

**Table 4:** Impact of treatment strategies on the primary outcome and its components according to the LVEF ranges and sensitivity analyses.

**AVI (within 6 months) vs. medical treatment**	**Univariate analysis of death or HHF HR (95% CI) and *P*-value**	**Multivariate analysis HR (95% CI) and** ***P*****-value**		
		**All–cause death or HHF**	**All–cause death**	**HHF**
Overall patients (*n* = 1,052)*				
LVEF ≤35%	0.74 (0.28–1.95) *P* = 0.543	0.65 (0.21–1.97) *P* = 0.441	0.54 (0.10–2.86) *P* = 0.468	0.71 (0.17–2.92) *P* = 0.630
LVEF 35–55%	0.24 (0.10–0.60) *P* = 0.002	0.15 (0.04–0.50) *P* = 0.002	0.05 (0.01–0.44) *P* = 0.006	0.18 (0.05–0.66) *P* = 0.010
LVEF >55%	0.38 (0.16–0.90) *P* = 0.028	0.40 (0.14–1.15) *P* = 0.089	0.34 (0.09–1.23) *P* = 0.099	0.53 (0.12–2.31) *P* = 0.397
Patients without CAD (*n* = 691)				
LVEF ≤35%	0.51 (0.15–1.75) *P* = 0.284	0.52 (0.12–2.25) *P* = 0.381	0.65 (0.12–3.42) *P* = 0.612	0.21 (0.02–1.92) *P* = 0.165
LVEF 35–55%	0.19 (0.06–0.63) *P* = 0.007	0.21 (0.05–0.86) *P* = 0.030	0.08 (0.01–0.61) *P* = 0.015	0.29 (0.05–1.62) *P* = 0.160
LVEF >55%	0.32 (0.11–0.91) *P* = 0.033	0.34 (0.11–1.04) *P* = 0.060	0.46 (0.12–1.75) *P* = 0.253	0.21 (0.03–1.61) *P* = 0.132
Patients without aortic disease (*n* = 881)				
LVEF ≤35%	0.63 (0.22–1.86) *P* = 0.406	0.55 (0.16–1.97) *P* = 0.361	0.46 (0.08–2.64) *P* = 0.386	0.61 (0.11–3.35) *P* = 0.572
LVEF 35–55%	0.24 (0.08–0.66) *P* = 0.006	0.10 (0.03–0.43) *P* = 0.002	Adjusted log–rank^†^ *P* = 0.007	0.15 (0.04–0.63) *P* = 0.010
LVEF >55%	0.38 (0.14–1.10) *P* = 0.074	0.41 (0.12–1.46) *P* = 0.169	0.29 (0.08–1.03) *P* = 0.056	0.63 (0.08–5.00) *P* = 0.658
Patients without secondary MR (*n* = 935)				
LVEF ≤35%	0.58 (0.16–2.03) *P* = 0.390	0.35 (0.09–1.47) *P* = 0.151	0.50 (0.10–2.69) *P* = 0.423	0.20 (0.02–1.91) *P* = 0.161
LVEF 35–55%	0.21 (0.07–0.58) *P* = 0.003	0.11 (0.03–0.37) *P* <0.001	0.05 (0.01–0.45) *P* = 0.007	0.12 (0.03–0.49) *P* = 0.003
LVEF >55%	0.45 (0.19–1.07) *P* = 0.070	0.45 (0.16–1.29) *P* = 0.139	0.30 (0.09–1.06) *P* = 0.062	0.63 (0.14–2.74) *P* = 0.535
Patients without other cardiac or aortic surgery (*n* = 886)^‡^				
LVEF ≤35%	1.18 (0.28–5.03) *P* = 0.819	1.18 (0.24–5.78) *P* = 0.834	1.04 (0.13–8.37) *P* = 0.97	1.10 (0.13–9.53) *P* = 0.933
LVEF 35–55%	0.17 (0.04–0.71) *P* = 0.015	0.03 (0.01–0.23) *P* = 0.001	0.048 (0.01–0.54) *P* = 0.014	0.02 (0.01–0.21) *P* = 0.001
LVEF >55%	0.37 (0.12–1.20) *P* = 0.099	1.67 (0.30–9.31) *P* = 0.562	0.13 (0.02–1.03) *P* = 0.054	3.28 (0.55–19.48) *P* = 0.191

*AVI, aortic valve intervention; CI, confidence interval; CAD, coronary artery disease; HHF, hospitalization for heart failure; HR, hazard ratio; LVEF, left ventricular ejection fraction; MR, mitral regurgitation*.

* *To avoid immortal–time bias, the time–zero was the time of aortic valve intervention for the recipients and day 15 following the baseline echocardiography for the non-recipients. In addition, only early aortic valve interventions performed within 6 months of the baseline echocardiography were evaluated in order to reduce the impact of changes in LVEF and AR severity during follow-up on assessment*.

† *Since no event had occurred in patients after aortic valve intervention within this LVEF range, the Cox model converged before the variable, resulting in an infinite coefficient; thus, adjusted log-rank test was adopted instead*.

‡ *Patients who underwent concomitant cardiac or aortic surgery during AVI procedures and patients who received isolated cardiac or aortic surgery without AVI were excluded*.

### Temporal Course of Symptom Status Under Different Treatment Strategies

**Figure 4:**
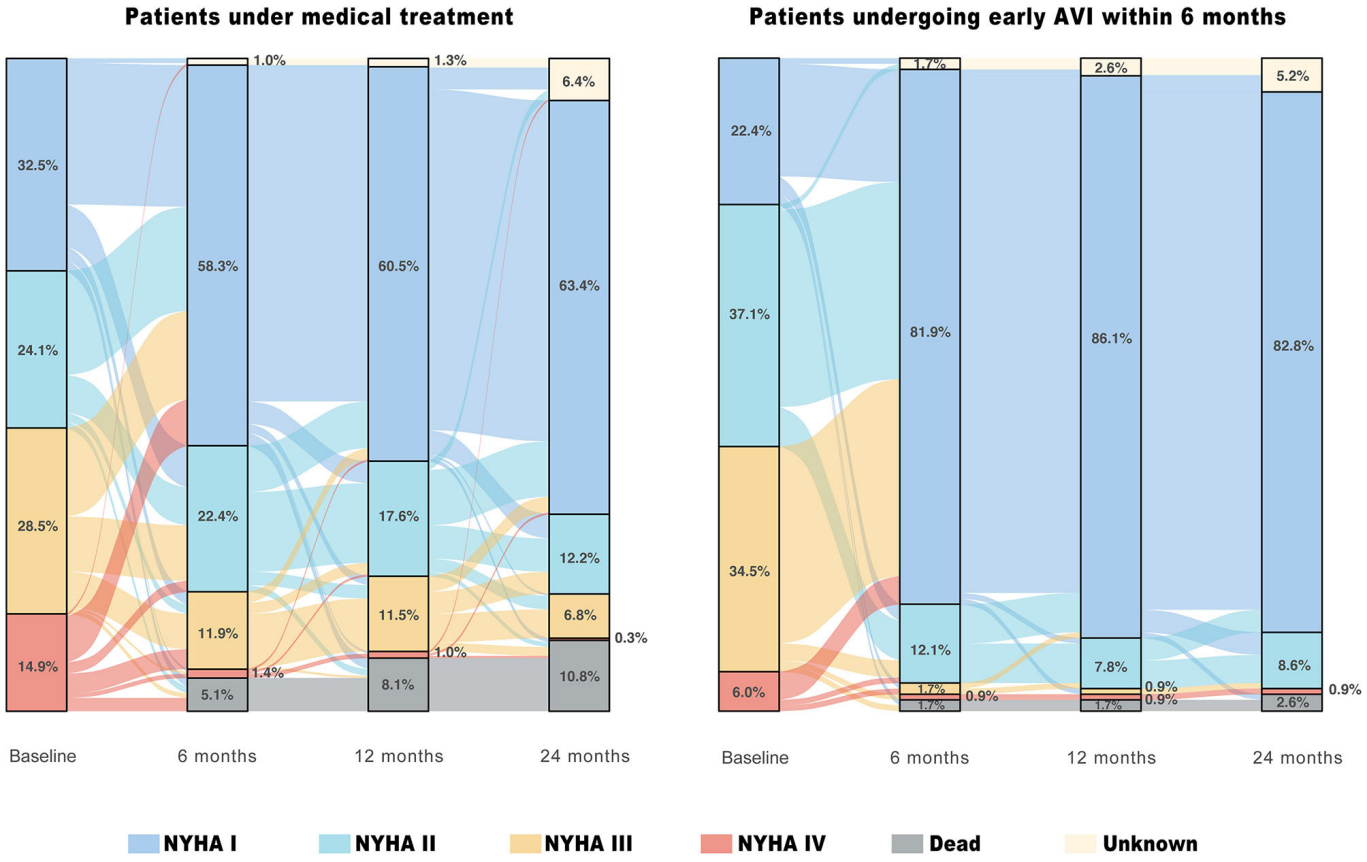
Temporal course of symptom status in patients with moderate AR and LVSD (LVEF ≤55%) under different treatment strategies. The river plots show the changes in New York Heart Association (NYHA) functional classification for patients under medical treatment who at least survived and were followed up for 15 days and for patients undergoing early aortic valve intervention (AVI) within 6 months of the baseline echocardiography.

Figure 4 shows the changes in the NYHA functional classification of patients with LVSD (LVEF ≤55%) under MT and early AVI treatment (within 6 months). At 6, 12, and 24 months, 58.3, 60.5, and 63.4% of patients under MT and 81.9, 86.1, and 82.8% of the patients undergoing early AVI had NYHA class I, respectively. At 2 years, among patients under MT and AVI treatment, 35.5 and 65.4% improved by at least one NYHA class compared to the baseline, 14.1 and 6.5% experienced symptom worsening or death, and 42.3 and 22.9% had no change in symptoms (*P* < 0.001), respectively.

## Discussion

Based on a large multicenter prospective cohort, this study was novel in its exploration of the prognostic value of LVEF in patients with moderate AR under different treatment strategies. Our key findings are as follows: (1) Reduced LVEF is the most contributive independent predictor of death or HHF in patients with moderate AR; (2) LVEF ≤55% was the optimal threshold for predicting poor prognosis under MT, with excellent performance in risk stratification and substantial incremental value; (3) At an LVEF of 35–55%, early AVI within 6 months of diagnosis was associated with a reduced risk of death or HHF vs. MT alone, whereas this prognostic benefit was markedly attenuated when LVEF decreased to ≤35% or increased to >55%. These findings suggest that LVEF, as a routine echocardiographic measure of LV systolic function, is crucial in risk stratification and provides potential implications for treatment decision-making in patients with moderate AR.

### Prevalence of Coexistent Moderate AR and LVSD

The coexistence of moderate AR and LVSD may be common in the general population but is underreported. A large population-based study enrolling 79,043 community-dwelling patients demonstrated moderate AR in 1.83% of patients with HF symptoms (4). Moreover, a retrospective epidemiological study showed that 24.4% of hospitalized patients with AR had HF (16). The present nationwide multicenter study, enrolling both inpatients and outpatients, first provided direct evidence that 38.6% of patients with moderate AR had reduced LVEF (≤55%), and 24.1% had NYHA class III/IV HF. Thus, the prevalence of coexistent moderate AR and LVSD should be of sufficient concern, since it is expected to rise with an aging population (1–3).

### Prognostic Impact of Coexistent Moderate AR and LVSD

Moderate AR is generally not considered a serious clinical issue in patients with normal LV systolic function. However, with LVSD, the presence of moderate AR may not be benign. Whether as a direct cause or a comorbid condition of LVSD, significant AR imposes a persistent hemodynamic burden on the failing LV and exacerbates systolic dysfunction (5). Additionally, significant AR also reduces coronary blood flow reserve (5). With LVSD, myocardial oxygen consumption increases due to the activation of compensatory mechanisms (17); however, intracoronary blood flow in patients with moderate AR may be insufficient to meet the increasing demand, thereby inducing subendocardial hypoxia and deteriorating LV function. In our study, when LVEF was >55%, the 2-year risk of death or HHF in patients with moderate AR under MT was relatively low and plateaued, whereas the risk increased sharply once LVEF fell below that level. LVEF ≤55% was the optimal risk-prediction threshold under MT, independently associated with a >2-fold increase in the 2-year risk of death or HHF. This threshold was higher than that for the age- and sex-matched population without left-sided VHD (LVEF ≤48%). Consistently, prior echocardiographic studies showed that in the general population, it is usually when LVEF drops to 45–50% that the risk of cardiovascular events begins to increase dramatically (18–20), suggesting the combination of moderate AR may lead to an earlier LV decompensation in patients with LVSD.

### Therapeutic Implications for Patients With Moderate AR and LVSD

Despite the common coexistence and poor prognosis of moderate AR and LVSD, the optimal treatment strategy remains unclear. Current AHA/ACC guidelines classify the management of patients with AR according to stages A to D (7). However, patients with moderate AR and LVSD cannot be categorized as stage B (mild/moderate AR with normal LVEF) or C/D (severe AR with normal/reduced LVEF ≤55%). Although recent evidence suggests that patients with moderate aortic stenosis and LVSD can benefit from AVI (9, 10), data on the effectiveness of AVI in patients with moderate AR and LVSD remain scarce.

Theoretically, among patients with LVSD, hemodynamic overload reduction in the form of mechanical relief from significant AR may substantially improve long-term prognosis (8). However, decision-making for AVI should carefully balance the prognostic benefits and risks. Although AVI can normalize the hemodynamics of moderate AR and contribute to systolic function recovery, it also exposes patients to prosthetic valve-related complications, such as anticoagulation-related bleeding, endocarditis, and prosthetic valve failure (21). More importantly, as LVEF decreases, the surgical risks increase correspondingly, whereas the prognostic benefits from AVI gradually diminish as LV remodeling progresses (22). Herein, we observed that among patients with moderate AR and LVSD, AVI was associated with a reduced risk of death or HHF at an LVEF of 35–55% compared with MT alone. The prognostic benefit of AVI was markedly attenuated when LVEF was ≤35%. Although previous studies reported that patients with severe AR and severe LVSD (LVEF ≤35%) could still derive substantial prognostic improvements from AVI with acceptable surgical risk (8), the benefits of surgical correction of moderate AR may not be as significant as that of severe AR and no longer outweigh the associated risks.

Notably, according to the current AHA/ACC guidelines, the decision to intervene in moderate AR depends largely on the need for other concomitant cardiac or aortic surgery (Class IIa, Level C), with the most frequent being CABG and aortic surgery (7). Half of the present cohort receiving AVI underwent concomitant surgery (51.3%). In this scenario, AVI serves mainly as prophylaxis in the treatment of moderate AR to avoid repeat open-heart surgery. This indication remains controversial in 2021 ESC/EACTS guidelines, as previous small single-center data showed that the progression of moderate AR is slow and indolent (6, 23). However, a recent large-sample investigation of the natural history of AR showed that the 10-year incidence of progression to stage C/D AR was 53.4% among patients with moderate AR (median 2.96 [1.2–5.4] years), in contrast to those with trivial/mild AR (11.7%, HR = 4.71) (24). Our findings further extend guideline indications by proposing that in addition to the prophylactic role, surgical correction of moderate AR can also translate to symptoms and outcomes improvements among patients with LVEF 35–55%. Furthermore, the findings remained consistent in patients without concomitant surgery and associated diseases, indicating that the prognostic improvements from AVI in patients with moderate AR and LVSD were independent of the surgical treatment for cardiac or aortic comorbidities.

### Study Limitations

First, as a nationwide multicenter study, echocardiographic data were site reported instead of core lab reported. However, to ensure diagnostic accuracy and measurement consistency, a series of quality control measures had been implemented (detailed in Supplementary Methods). All participating centers were instructed to have experienced sonographers perform echocardiography according to the specific guidelines (12). AR severity was graded using an integrative approach and reviewed by senior physicians or surgeons (11). All echocardiographic records were sent to the coordinating center for inspection. Randomly sampled images were gathered from each center and blindly reviewed at the core lab in Fuwai Hospital.

Second, due to the limitation of routine clinical echocardiography, we could not collect detailed AR-specific quantitative data from all centers; therefore, regurgitant volume and regurgitation orifice area were not systematically used to construct prediction models. However, this limitation may not hamper the evaluation of the prognostic value of LVEF in patients with moderate AR. Unlike severe AR, these quantitative parameters in moderate AR were defined within a restricted range. Thus, the prognostic impact attributable to parameter variation may be limited, as evidenced by a large-sample study of patients with stage B AR, wherein none of these AR-specific quantitative parameters were independent prognostic determinants (24). Also, our models achieved satisfactory predictive performance based on the available variables.

Third, SAVR remains the first-line treatment for AR in current practice, accounting for over 90% of the AVI in this cohort. Whether TAVR, as a minimally invasive approach to correct AR, can achieve better clinical outcomes in patients with moderate AR and severe LVSD (LVEF ≤35%) requires prospective evaluations.

Finally, as an observational study, treatments were not randomly assigned. Although we used IPTW to reduce the inherent bias, unmeasured confounding factors may exist. Thus, our findings warrant further evaluation in a randomized setting.

### Conclusions

LVEF is an independent and incremental prognostic factor in patients with moderate AR, and LVEF ≤55% is a robust marker of poor prognosis under MT. At an LVEF of 35–55%, surgical correction of moderate AR within 6 months of diagnosis is associated with substantial symptoms and outcomes improvements compared with MT alone. As an easily accessible echocardiographic index, LVEF plays a crucial role in risk stratification and provides potential implications for treatment decision-making in patients with moderate AR.

## Data Availability Statement

The raw data supporting the conclusions of this article will be made available by the authors, without undue reservation.

## Ethics Statement

The studies involving human participants were reviewed and approved by Institutional Review Boards at the National Center for Cardiovascular Diseases of China. The patients/participants provided their written informed consent to participate in this study.

## Author Contributions

YW and RG conceived the study. QZ, HX, BZ, and RZ developed the study design and methodology. YY, ZL, QL, ZZ, WW, ZY, HZ, ZD, BW, JL, and SG were involved in the study implement, data collection, and data audit. QZ, BZ, and YZ conducted statistical analyses, interpreted the results, and edited the initial draft of the manuscript. YW, HX, BZ, YY, and ZL critically revised the manuscript for important intellectual content. All authors contributed to the article and approved the submitted version.

## Funding

This study was supported by the Chinese Academy of Medical Sciences Innovation Fund for Medical Sciences (2017-12M-3-002).

## Conflict of Interest

The authors declare that the research was conducted in the absence of any commercial or financial relationships that could be construed as a potential conflictof interest.

## Publisher's Note

All claims expressed in this article are solely those of the authors and do not necessarily represent those of their affiliated organizations, or those of the publisher, the editors and the reviewers. Any product that may be evaluated in this article, or claim that may be made by its manufacturer, is not guaranteed or endorsed by the publisher.

## Abbreviations

AR: aortic regurgitation
LVSD: left ventricular systolic dysfunction
LVEF: left ventricular ejection fraction
AVI: aortic valve intervention
MT: medical treatment
HF: heart failure
HHF: hospitalization for heart failure.
