# Supplementary material for: Prognostic Impact of Left Ventricular Ejection Fraction in Patients With Moderate Aortic Regurgitation: Potential Implications for Treatment Decision-Making
Source: Front Cardiovasc Med. 2022 Jan 17;8:800961. doi: 10.3389/fcvm.2021.800961 (PMC8802721; doi:10.3389/fcvm.2021.800961)

SUPPLEMENTARY MATERIAL

Zhao et al. Prognostic Impact of Left Ventricular Ejection Fraction in Patients with Moderate Aortic Regurgitation: Potential Implications for Treatment Decision-Making

Contents:

I. Supplementary methods

Data collection, management, and data quality control in the China-VHD study **(P4)**

Quality control of echocardiographic measurement in the China-VHD study **(P5)**

Statistical analyses **(P5-8)**

II. Supplementary references (P9-11)

III. Supplementary Tables

Supplementary Table 1: Number of missing values and corresponding dispositions **(P12)**

Supplementary Table 2: Baseline characteristics of age- and sex-matched population without left-sided valvular heart disease **(P13-14)**

Supplementary Table 3: Univariable analysis of predictive factors of the composite of death or HHF under medical treatment **(P15-16)**

Supplementary Table 4: Univariable analysis of predictive factors of the composite of death or HHF under aortic valve intervention treatment **(P17-18)**

Supplementary Table 5: Subgroup analyses of the association between baseline LVEF and the risk of death or HHF under medical treatment **(P19)**

Supplementary Table 6: Assessment of the impact of treatment strategies on the composite of death or HHF according to the LVEF ranges using the IPTW Cox regression model and the time-dependent Cox regression model based on the entire cohort **(P20-21)**

Supplementary Table 7: Assessment of the impact of treatment strategies on the composite of death or HHF according to the LVEF ranges and reasons of admission **(P22)**

IV. Supplementary Figure

Supplementary Figure Legend (P23-26)

Supplementary Figure 1: Hospitals participating in the China-VHD study and their geographical distribution. **(P27-29)**

Supplementary Figure 2: Flow chart of the study cohort. **(P30)**

Supplementary Figure 3: Variable selection for predicting death or HHF under medical treatment performed by the LASSO-penalized Cox regression and the coefficients of the selected variables. **(P31)**

Supplementary Figure 4: Relative importance of the predictors of death or HHF under medical treatment selected by the LASSO-penalized Cox regression**. (P32)**

Supplementary Figure 5: Variable selection for predicting death or HHF under aortic valve intervention treatment performed by the LASSO-penalized Cox regression and the coefficients of the selected variables. **(P33)**

Supplementary Figure 6: Relative importance of the predictors of death or HHF under aortic valve intervention treatment selected by the LASSO-penalized Cox regression. **(P34)**

Supplementary Figure 7: Determination of the best LVEF cutoff value for predicting death or HHF under medical treatment using the maximally selected rank statistics method and the relative risk with the selected cut-point as reference. **(P35)**

Supplementary Figure 8: Decision curve analysis of the risk-prediction models with and without baseline LVEF for the composite of death or HHF under medical treatment. **(P36)**

Supplementary Figure 9: Association between baseline LVEF and relative hazard of 2-year death or HHF in age- and sex-matched population without left-sided valvular heart disease. **(P37)**

Supplementary Figure 10: Determination of the best LVEF cutoff value for predicting death or HHF in age- and sex-matched population without left-sided valvular heart disease using the maximally selected rank statistics method, and the relative risk with the selected cut-point as reference, and Kaplan-Meier curves of event-free survival. **(P38)**

Supplementary Figure 11: Graphical inspection of scaled Schoenfeld residuals to test the proportional hazard assumption. **(P39)**

Supplementary Figure 12. Absolute standardized mean differences across covariates before and after the inverse probability of treatment weighted adjustment. **(P40)**

I. Supplementary methods

Data collection, management, and data quality control in the China-VHD study

In the China-VHD study, comprehensive data with standard definitions were collected, validated, and submitted through a web-based electronic data capture (EDC) system, including presentation, investigations, treatments, and outcomes. All site investigators received detailed training on the protocols and data collection. Enrollment, data collection, and follow-up were performed by trained cardiology fellows, cardiologists, or cardiothoracic surgeons at each site in a real-time manner to ensure data accuracy and reliability. Site investigators were instructed to collect the baseline data during the hospitalization and complete all required information in the EDC system upon the patients' discharge or death. Follow-up data were obtained from patient visits, medical records, and telephone interviews. Death, hospitalization, and intervention reports were validated by investigators at each center. Appointed and trained local senior cardiologists and cardiothoracic surgeons were responsible for the quality of data collection at their own hospitals (**Supplementary Figure 1**).

Periodic database checking was undertaken. The data management team regularly provided data quality checks and sent queries for illogical, invalid, or missing data elements to participating sites to review and revise.

Randomly sampled hospital sites received on-site audits. Trained auditors went through medical records of the hospitals during the enrollment period to evaluate the consecutiveness of enrollment. They also reviewed medical records of patients drawn randomly to check the accuracy of diagnosis, compliance with the inclusion criteria, and consistency with the submitted data.

Quality control of echocardiographic measurement in the China-VHD study

A standard echocardiography protocol was provided to operators and reporters at participating sites. Experienced sonographers at each center (**Supplementary Figure 1**) were instructed to perform clinical echocardiography and to grade VHD severity according to the specific guidelines and standard protocol.[1,2] Echocardiographic records were then reviewed by the senior cardiologists and cardiothoracic surgeons at the same center to ensure diagnostic accuracy and compliance with the inclusion criteria. Before starting the recruitment process, training workshops were held for the participating sonographers to ensure diagnostic accuracy and measurement consistency, during which sample images were analyzed and feedback was provided by echo experts to improve the quality. During the enrollment, all echocardiographic records must be sent to the study coordinating center as resource files for inspection. Randomly sampled images were gathered from each center and were blindly reviewed to verify diagnostic accuracy and measurement consistency at the core lab in Fuwai Hospital, National Center for Cardiovascular Disease.

Statistical analysis

Missing values were imputed using the multiple imputations. Detailed information on the numbers of missing values and descriptive statistics are shown in **Supplementary Table 1**. The proportional hazard assumptions of the Cox proportional-hazards models were verified by inspecting the Schoenfeld residuals (**Supplementary Figure 10**).

To assessed the impact of the presence of moderate AR on the association between LVEF and the primary outcome, we further assessed the association pattern in an age- and sex-matched population without left-sided valvular heart disease for comparison (**Supplementary Figure 8**). This population was derived from the present cohort using the propensity score matching method, with the exclusion of those with previous valvular intervention, active endocarditis, ≥moderate aortic or mitral stenosis, ≥moderate aortic or mitral regurgitation,

dilated and hypertrophic cardiomyopathy, congenital heart disease, acute aortic syndrome, aortic rupture, and acute myocardial infarction within 90 days. Matching was performed using a 1:1 greedy matching protocol without replacement, with a caliper width equal to 0.1 of the standard deviation of the logit of the propensity score. Finally, 1211 age- and sex-matched patients without left-sided valvular heart disease were included (**Supplementary Table 2**), with 112 (9.2%) death or hospitalization for heart failure (HHF) occurred during the 2-year follow-up. Among them, we employed the maximally selected rank statistics method to determine the most significant LVEF cutoff for predicting death or HHF (**Supplementary Figure 9A**). Based on the selected LVEF threshold, we estimated the cumulative incidences of outcomes using the Kaplan-Meier method and assessed hazard ratios with 95% confidence intervals using Cox proportional-hazards models (**Supplementary Figure 9C**).

In the multivariate analysis, we employed an integrative approach to select variables associated with the primary outcome under medical treatment and aortic valve intervention (AVI), respectively. We initially examined all baseline variables by univariate analyses (**Supplementary Table 3; Supplementary Table 4**). Then, the least absolute shrinkage and selection operator (LASSO)-penalized Cox regression models were performed to identify the variables associated with the primary outcome,[3] where baseline variables with P <0.25 in the univariate analyses or clinically relevance were entered (**Supplementary Figure 2, Supplementary Figure 4**). LASSO algorithm is a widely recommended machine-learning method for predictive variable selection that has been proven advantageous over traditional regression methods.[4] To maximize the predictive power and ensure parsimony of the model, the tuning parameter λ selection in the LASSO model used 10-fold cross-validation via minimum criteria. The relative importance of the selected predictors was ranked and validated based on the proportion of explainable log-likelihood ratio χ^2 statistics (**Supplementary Figure 3, Supplementary Figure 5**).[5]

In the analysis assessing the impact of AVI on the primary outcome according to the different LVEF ranges, the inverse probability of treatment weighted (IPTW) Cox regression model was used to account for bias due to non-random treatment assignment [6]. The propensity scores used for IPTW were estimated using the multivariate logistic model where AVI treatment was the dependent variable, and plausible correlates of either AVI or the primary outcome acted as independent variables.[7] To avoid unnecessary adjustment leading to model over-fitting, the variables used to construct IPTW weights were selected based on the findings of the LASSO model abovementioned and previous studies,[8–12] with additional regard for clinical relevance, thus using age, sex, body mass index, systolic blood pressure, coronary artery disease, prior myocardial infarction, prior coronary artery bypass grafting, atrial fibrillation, aortic disease, chronic kidney disease, New York Heart Association (NYHA) class III/IV, hemoglobin, left ventricular ejection fraction, left ventricular end-systolic diameter >50 mm, moderate secondary mitral regurgitation, pulmonary hypertension, EuroSCORE-II, use of angiotensin-converting enzyme inhibitors/angiotensin receptor blockers (ACEI/ARB) and use of beta-blocker to construct IPTW weights. In the main text, to avoid immortal-time bias and reduce the impact of changes in LVEF and AR severity during follow-up on the assessment,[13] the analysis was based on patients under medical treatment who at least survived or were followed up for 15 days and on patients under early AVI treatment who underwent the procedure within 6 months of the baseline echocardiography. To ensure the robustness of our findings, we added sensitivity analyses to assess the impact of AVI on the primary outcome based on the entire cohort using the same IPTW Cox regression model (**Supplementary Table 6**). Also, multivariable Cox proportional-hazard model was constructed based on the entire cohort, where AVI was treated as a time-dependent covariate, adjusted for age, sex, body mass index, coronary artery disease, prior myocardial infarction, prior coronary artery bypass grafting, atrial fibrillation, chronic kidney disease, NYHA class III/IV, left ventricular ejection

fraction, pulmonary hypertension, EuroSCORE-II, use of ACEI/ARB and use of beta-blocker (**Supplementary Table 6**).

II. Supplementary references

1. Nishimura RA, Otto CM, Bonow RO, Carabello BA, Erwin JP, Guyton RA, O'Gara PT, Ruiz CE, Skubas NJ, Sorajja P, Sundt TM, Thomas JD. 2014 AHA/ACC guideline for the management of patients with valvular heart disease. *J Am Coll Cardiol* 2014;**63**:57–185.
2. Lang RM, Badano LP, Victor MA, Afilalo J, Armstrong A, Ernande L, Flachskampf FA, Foster E, Goldstein SA, Kuznetsova T, Lancellotti P, Muraru D, Picard MH, Retzschel ER, Rudski L, Spencer KT, Tsang W, Voigt JU. Recommendations for cardiac chamber quantification by echocardiography in adults: An update from the American Society of Echocardiography and the European Association of Cardiovascular Imaging. *J Am Soc Echocardiogr* Elsevier Inc; 2015;**28**:1-39.e14.
3. Tibshirani R. Regression shrinkage and selection via the lasso. *J R Stat Soc Ser B* Wiley Online Library; 1996;**58**:267–288.
4. Goldstein BA, Navar AM, Carter RE. Moving beyond regression techniques in cardiovascular risk prediction: Applying machine learning to address analytic challenges. *Eur Heart J* 2017;**38**:1805–1814.
5. Beyene J, Atenafu EG, Hamid JS, To T, Sung L. Determining relative importance of variables in developing and validating predictive models. *BMC Med Res Methodol* BioMed Central; 2009;**9**:1–10.
6. Haukoos JS, Lewis RJ. The propensity score. *JAMA - J Am Med Assoc* 2015;**314**:1637–1638.
7. Rubin DB, Thomas N. Matching using estimated propensity scores: relating theory to practice. *Biometrics* JSTOR; 1996;249–264.

8. Yang LT, Enriquez-Sarano M, Michelena HI, Nkomo VT, Scott CG, Bailey KR, Oguz D, Wajih Ullah M, Pellikka PA. Predictors of Progression in Patients With Stage B Aortic Regurgitation. *J Am Coll Cardiol* 2019;**74**:2480–2492.

9. Meester C de, Gerber BL, Vancraeynest D, Pouleur AC, Noirhomme P, Pasquet A, Kerchove L de, Khoury G El, Vanoverschelde JL. Do Guideline-Based Indications Result in an Outcome Penalty for Patients With Severe Aortic Regurgitation? *JACC Cardiovasc Imaging* 2019;**12**:2126–2138.

10. Murashita T, Schaff H V., Suri RM, Daly RC, Li Z, Dearani JA, Greason KL, Nishimura RA. Impact of Left Ventricular Systolic Function on Outcome of Correction of Chronic Severe Aortic Valve Regurgitation: Implications for Timing of Surgical Intervention. *Ann Thorac Surg* The Society of Thoracic Surgeons; 2017;**103**:1222–1228.

11. Mentias A, Feng K, Alashi A, Rodriguez LL, Gillinov AM, Johnston DR, Sabik JF, Svensson LG, Grimm RA, Griffin BP, Desai MY. Long-Term Outcomes in Patients With Aortic Regurgitation and Preserved Left Ventricular Ejection Fraction. *J Am Coll Cardiol* 2016;**68**:2144–2153.

12. Yancy CW, Jessup M, Bozkurt B, Butler J, Casey DE, Colvin MM, Drazner MH, Filippatos GS, Fonarow GC, Givertz MM, Hollenberg SM, Lindenfeld JA, Masoudi FA, McBride PE, Peterson PN, Stevenson LW, Westlake C. 2017 ACC/AHA/HFSA Focused Update of the 2013 ACCF/AHA Guideline for the Management of Heart Failure: A Report of the American College of Cardiology/American Heart Association Task Force on Clinical Practice Guidelines and the Heart Failure Society of Amer. *J Am Coll Cardiol* 2017;**70**:776–803.

13. Zhou Z, Rahme E, Abrahamowicz M, Pilote L. Survival bias associated with time-to-treatment initiation in drug effectiveness evaluation: A comparison of methods. *Am J Epidemiol* 2005;**162**:1016–1023.

III. Supplementary tables

Supplementary Table 1: Number of missing values and corresponding dispositions

Variables	Number of missing values	Disposition
Systolic blood pressure	3 (0.2%)	multiple imputation
Diastolic blood pressure	3 (0.2%)	multiple imputation
Heart rate	3 (0.2%)	multiple imputation
Hemoglobin	14 (1.1%)	multiple imputation
eGFR	10 (0.8%)	multiple imputation
LDL	76 (6.3%)	multiple imputation
Total cholesterol	72 (5.9%)	multiple imputation
Ascending aortic diameter	10 (0.8%)	multiple imputation

Abbreviations: eGFR= estimated glomerular filtration rate; LDL= low-density lipoproteins;

Supplementary Table 2: Baseline characteristics of age- and sex-matched population without left-sided valvular heart disease

Characteristic	Study population (n=1211)	Matched population (n=1211)	P value
**Demographics and vital signs**			
Age, yr [Median (IQR)]	66 (57-73)	65 (53-74)	0.058
Male, no. (%)	819 (67.6%)	777 (64.2%)	0.079
BMI, kg/m^2 [Median (IQR)]	23.8 (21.5-26.1)	23.2 (21.0-25.3)	<0.001
BSA, m^2 (Mean±SD)	1.83±0.19	1.82±0.22	<0.001
Heart rate, bpm [Median (IQR)]	72 (65-81)	76 (65-88)	<0.001
SBP, mmHg [Median (IQR)]	130 (120-144)	123 (112-139)	<0.001
DBP, mmHg [Median (IQR)]	73 (65-80)	75 (67-84)	<0.001
**Risk factors and comorbidities**			
Current Smoker, no. (%)	234 (19.3%)	188 (15.5%)	0.786
Hypertension, no. (%)	719 (59.4%)	521 (43.0%)	<0.001
Diabetes, no. (%)	153 (12.6%)	174 (14.4%)	0.212
Dyslipidemia, no. (%)	196 (16.2%)	123 (10.2%)	<0.001
Atrial fibrillation, no. (%)	221 (18.2%)	457 (37.7%)	<0.001
Coronary artery disease, no. (%)	500 (41.3%)	396 (32.7%)	<0.001
Prior Myocardial infarction, no. (%)	78 (6.4%)	78 (6.4%)	1.000
Prior PCI, no. (%)	166 (13.7%)	108 (8.9%)	<0.001
Prior CABG, no. (%)	19 (1.6%)	33 (2.7%)	0.067
Aortic disease, no. (%)	178 (14.7%)	70 (5.8%)	<0.001
Cerebrovascular disease, no. (%)	134 (11.1%)	123 (10.2%)	0.509
Peripheral artery disease, no. (%)	53 (4.4%)	33 (2.7%)	0.027
Chronic obstructive pulmonary disease, no. (%)	74 (6.1%)	118 (9.7%)	0.001
Chronic kidney disease, no. (%)	72 (5.9%)	67 (5.5%)	0.662
**Baseline Symptoms**			
Dyspnea, no. (%)	619 (51.1%)	636 (52.5%)	0.489
Chest pain, no. (%)	332 (27.4%)	222 (18.3%)	<0.001
NYHA functional class, no. (%)			<0.001
I	560 (46.2%)	610 (50.4%)	

II	359 (29.6%)	239 (19.7%)	
III	217 (17.9%)	250 (20.6%)	
IV	75 (6.2%)	112 (9.2%)	
**Laboratory**			
Hemoglobin, g/L [Median (IQR)]	134 (121-146)	135 (120-150)	0.193
eGFR, ml/min/1.73m2 (Mean±SD)	85.3±26.8	83.9±33.3	0.091
LDL, mmol/L [Median (IQR)]	2.3 (1.8-2.9)	2.1 (1.6-2.7)	<0.001
Total cholesterol, mmol/L [Median (IQR)]	4.0 (3.3-4.7)	3.7 (3.1-4.4)	<0.001
NT-pro BNP, pg/ml [Median (IQR)]	539 (150-2105)	1207 (423-2844)	<0.001
BNP, pg/ml [Median (IQR)]	49 (19-160)	193 (113-536)	<0.001
**Echocardiography**			
LVEF, % [Median (IQR)]	59 (52-64)	60 (53-64)	0.309
LVESD, mm [Median (IQR)]	36 (31-42)	31 (27-36)	<0.001
LVESDi, mm/m^2 [Median (IQR)]	19.5 (17.2-23.5)	18.8 (16.6-22.2)	<0.001
LVEDD, mm [Median (IQR)]	54 (49-60)	47 (42-52)	<0.001
LVEDDi, mm /m^2 [Median (IQR)]	29.8 (26.9-33.4)	27.9 (25.3-31.6)	<0.001
LAi, mm/m^2 [Median (IQR)]	21.7 (19.3-25.0)	24.9 (21.8-28.7)	<0.001
Pulmonary hypertension, no.(%)	236 (19.5%)	281 (23.2%)	0.026
Ascending aortic diameter, mm	36 (31-41)	32 (29-35)	<0.001

Abbreviations: BMI= body mass index; BNP= B-type natriuretic peptide; BSA= body surface area; CABG= coronary artery bypass grafting; DBP= diastolic blood pressure; eGFR= estimated glomerular filtration rate; EuroSCORE-II= European System for Cardiac Operative Risk Evaluation; IQR= interquartile range; LA= left atrium end-diastolic dimension; LDL= low-density lipoproteins; LVEDD= left ventricular end-diastolic dimension; LVEF =left ventricular ejection fraction; LVESD= left ventricular end-systolic dimension; MR= mitral regurgitation; NT-proBNP= N-terminal pro-B-type natriuretic peptide; NYHA= New York Heart Association; PCI= percutaneous coronary intervention; SBP= systolic blood pressure; TR= tricuspid regurgitation;

Supplementary Table 3: Univariable analysis of predictive factors of the composite of death or HHF under medical treatment.

Variables	HR (95% CI)	P value
**Demographics and vital signs**		
Age	1.047 (1.028-1.067)	<0.001
Male	0.842 (0.574-1.237)	0.382
BMI	0.905 (0.857-0.956)	<0.001
BSA	0.093 (0.034-0.256)	<0.001
Heart rate	1.013 (1.003-1.022)	0.010
SBP	0.993 (0.984-1.002)	0.131
DBP	0.979 (0.964-0.994)	0.006
**Risk factors and comorbidities**		
Current Smoker	0.716 (0.415-1.235)	0.230
Hypertension	1.036 (0.703-1.527)	0.857
Diabetes	0.871 (0.505-1.503)	0.621
Dyslipidemia	0.565 (0.295-1.083)	0.086
Atrial fibrillation	2.117 (1.424-3.148)	<0.001
Coronary artery disease	1.015 (0.697-1.479)	0.939
Prior Myocardial infarction	2.037 (1.180-3.514)	0.011
Prior PCI	0.969 (0.584-1.607)	0.903
Prior CABG	3.812 (1.771-8.205)	0.001
Aortic disease	1.333 (0.828-2.146)	0.237
Cerebrovascular disease	1.088 (0.640-1.850)	0.755
Peripheral artery disease	0.615 (0.226-1.668)	0.339
Chronic obstructive pulmonary disease	1.601 (0.879-2.918)	0.124
Chronic kidney disease	2.964 (1.787-4.914)	<0.001
**Baseline Symptoms**		
Dyspnea	1.637 (1.135-2.360)	0.008
Chest pain	0.970 (0.649-1.450)	0.882
NYHA class III/IV	2.827 (1.936-4.127)	<0.001
**Laboratory**		

Hemoglobin	0.978 (0.970-0.987)	<0.001
eGFR	0.973 (0.963-0.983)	<0.001
LDL	0.754 (0.592-0.960)	0.022
Total cholesterol	0.776 (0.645-0.934)	0.007
Ln NT-pro BNP	1.600 (1.392-1.838)	<0.001
Ln BNP	1.370 (0.988-1.899)	0.059
**Echocardiography**		
LVEF	0.960 (0.948-0.972)	<0.001
LVESD	1.028 (1.011-1.044)	0.001
LVESD >50 mm	1.755 (1.080-2.852)	0.023
LVESDi	1.073 (1.045-1.101)	<0.001
LVEDD	1.016 (0.996-1.036)	0.109
LVEDD >70 mm	1.405 (0.710-2.779)	0.329
LVEDDi	1.070 (1.039-1.102)	<0.001
LAi	1.111 (1.076-1.147)	<0.001
Dysmorphic aortic valve (Unicuspid / Bicuspid/ Quadricuspid)	0.934 (0.380-2.292)	0.881
Moderate secondary MR	2.147 (1.381-3.336)	0.001
≥moderate TR	2.209 (1.473-3.313)	<0.001
Pulmonary hypertension	3.427 (2.350-5.000)	<0.001
Ascending aortic diameter	0.980 (0.952-1.009)	0.174
Ascending aortic diameter >45mm	0.978 (0.455-2.105)	0.956
**Admission for non-VHD related reasons**	0.450 (0.164-1.240)	0.123
**EuroSCORE-II**	1.222 (1.163-1.284)	<0.001

Abbreviations: BMI= body mass index; BNP= B-type natriuretic peptide; BSA= body surface area; CABG= coronary artery bypass grafting; DBP= diastolic blood pressure; eGFR= estimated glomerular filtration rate; EuroSCORE-II= European System for Cardiac Operative Risk Evaluation; IQR= interquartile range; LA= left atrium end-diastolic dimension; LDL= low-density lipoproteins; LVEDD= left ventricular end-diastolic dimension; LVEF =left ventricular ejection fraction; LVESD= left ventricular end-systolic dimension; MR= mitral regurgitation; NT-proBNP= N-terminal pro-B-type natriuretic peptide; NYHA= New York Heart Association; PCI= percutaneous coronary intervention; SBP= systolic blood pressure; TR= tricuspid regurgitation;

Supplementary Table 4: Univariable analysis of predictive factors of the composite of death or HHF under AVI treatment.

Variables	HR (95% CI)	P value
**Demographics and vital signs**		
Age	1.059 (1.008-1.114)	0.024
Male	1.986 (0.451-8.738)	0.364
BMI	0.836 (0.707-0.988)	0.035
BSA	0.228 (0.013-4.092)	0.316
Heart rate	0.996 (0.958-1.035)	0.832
SBP	0.994 (0.968-1.022)	0.688
DBP	0.992 (0.948-1.038)	0.726
**Risk factors and comorbidities**		
Current Smoker	0.846 (0.241-2.969)	0.794
Hypertension	0.697 (0.260-1.871)	0.474
Diabetes	4.531 (1.030-19.937)	0.046
Dyslipidemia	0.316 (0.042-2.390)	0.264
Atrial fibrillation	1.702 (0.387-7.489)	0.482
Coronary artery disease	2.576 (0.959-6.919)	0.060
Prior Myocardial infarction	3.759 (0.853-16.569)	0.080
Prior PCI	2.725 (0.619-11.994)	0.185
Prior CABG	12.929 (1.698-98.441)	0.013
Aortic disease	1.417 (0.457-4.392)	0.546
Cerebrovascular disease	7.761 (2.500-24.092)	<0.001
Peripheral artery disease	0 (0-Inf)	0.998
Chronic obstructive pulmonary disease	1.918 (0.253-14.518)	0.528
Chronic kidney disease	0 (0-Inf)	0.997
**Baseline Symptoms**		
Dyspnea	0.924 (0.321-2.660)	0.884
Chest pain	0.713 (0.203-2.502)	0.597
NYHA class III/IV	1.438 (0.523-3.958)	0.482
**Laboratory**		

Hemoglobin	1.004 (0.975-1.033)	0.809
eGFR	0.980 (0.953-1.008)	0.166
LDL	0.857 (0.465-1.579)	0.621
Total cholesterol	0.822 (0.499-1.355)	0.443
Ln NT-pro BNP	1.338 (0.899-1.989)	0.151
Ln BNP	Inf (0-Inf)	0.998
**Echocardiography**		
LVEF	0.587 (0.389-0.887)	0.011
LVESD	1.027 (0.976-1.081)	0.297
LVESD >50 mm	1.616 (0.461-5.673)	0.454
LVESDi	1.050 (0.966-1.142)	0.250
LVEDD	1.008 (0.950-1.070)	0.786
LVEDD >70 mm	2.877 (0.819-10.105)	0.099
LVEDDi	1.027 (0.936-1.128)	0.570
LAi	1.084 (0.988-1.190)	0.090
Dysmorphic aortic valve (Unicuspid / Bicuspid/ Quadricuspid)	1.538 (0.349-6.766)	0.569
Moderate secondary MR	3.886 (1.107-13.637)	0.034
≥moderate TR	1.377 (0.182-10.425)	0.757
Pulmonary hypertension	2.529 (0.815-7.841)	0.108
Ascending aortic diameter	0.975 (0.915-1.038)	0.423
Ascending aortic diameter >45mm	1.145 (0.369-3.551)	0.814
**Admission for non-VHD related reasons**	0.971 (0.608-1.552)	0.902
**EuroSCORE-II**	1.461 (1.125-1.896)	0.004

Abbreviations: BMI= body mass index; BNP= B-type natriuretic peptide; BSA= body surface area; CABG= coronary artery bypass grafting; DBP= diastolic blood pressure; eGFR= estimated glomerular filtration rate; EuroSCORE-II= European System for Cardiac Operative Risk Evaluation; IQR= interquartile range; LA= left atrium end-diastolic dimension; LDL= low-density lipoproteins; LVEDD= left ventricular end-diastolic dimension; LVEF =left ventricular ejection fraction; LVESD= left ventricular end-systolic dimension; MR= mitral regurgitation; NT-proBNP= N-terminal pro-B-type natriuretic peptide; NYHA= New York Heart Association; PCI= percutaneous coronary intervention; SBP= systolic blood pressure; TR= tricuspid regurgitation;

Supplementary Table 5: Subgroup analyses of the association between baseline LVEF and the risk of death or HHF under medical treatment

Subgroup		LVEF ≤55% vs. LVEF >55% Unadjusted HR (95% CI) and P	LVEF ≤55% vs. LVEF >55% Adjusted HR (95% CI) and P *	P for interaction
**Age**	≥65 years	2.59 (1.66-4.04) P<0.001	1.88 (1.12-3.15) P=0.017	0.121
	<65 years	5.37 (2.27-12.70) P<0.001	3.18 (1.13-8.92) P=0.028	
**Sex**	Male	4.14 (2.38-7.18) P<0.001	3.19 (1.74-5.86) P<0.001	0.106
	Female	2.42 (1.33-4.40) P=0.004	1.95 (1.05-3.60) P=0.034	
**Symptoms**	NYHA II-IV	2.44 (1.46-4.09) P=0.001	1.80 (1.01-3.20) P=0.045	0.217
	NYHA I	3.25 (1.69-6.24) P<0.001	3.58 (1.70-7.57) P=0.001	
**CAD**	Yes	2.97 (1.66-5.32) P<0.001	2.58 (1.36-4.89) P=0.004	0.844
	No	3.30 (1.93-5.64) P<0.001	2.15 (1.19-3.89) P=0.011	
**Secondary MR**	Yes	2.40 (1.51-3.83) P<0.001	1.91 (1.10-3.32) P=0.022	0.121
	No	4.45 (2.13-9.30) P<0.001	2.68 (1.17-6.13) P=0.020	
**AR etiology**	Secondary AR	2.33 (1.27-4.29) P=0.007	2.21 (1.17-4.17) P=0.014	0.352
	Non-secondary AR	3.80 (2.27-6.39) P<0.001	2.54 (1.40-4.60) P=0.002	
**EuroSCORE-II**	>3	3.37 (1.40-8.11) P=0.007	3.16 (1.22-8.16) P=0.018	0.173
	≤3	2.46 (1.55-3.90) P<0.001	2.05 (1.22-3.46) P=0.007	

Abbreviations: AR= aortic regurgitation; CAD= coronary artery disease; CI= confidence interval; HR= hazard ratio; LVEF= left ventricular ejection fraction; MR= mitral regurgitation

* Adjusting for age, body mass index, atrial fibrillation, prior myocardial infarction, prior coronary artery bypass grafting, chronic kidney disease, New York Heart Association class III/IV, hemoglobin, left ventricular end-systolic diameter >50 mm, pulmonary hypertension, and EuroSCORE-II.

Supplementary Table 6: Assessment of the impact of treatment strategies on the composite of death or HHF according to the LVEF ranges using the IPTW Cox regression model and the time-dependent Cox regression model based on the entire cohort

Aortic valve intervention versus medical treatment	IPTW Cox regression model based on the entire cohort (n=1211) * for the composite of death or HHF		Time-dependent Cox regression model based on the entire cohort (n=1211) † for the composite of death or HHF	
	**Univariate analysis** HR (95% CI); P value	**Multivariate analysis** HR (95% CI); P value	**Univariate analysis** HR (95% CI); P value	**Multivariate analysis** HR (95% CI); P value
**Overall patients**				
LVEF ≤ 35%	0.69 (0.26-1.80) P=0.442	0.57 (0.20-1.67) P=0.305	0.76 (0.29-1.99) P=0.578	1.53 (0.50-4.69) P=0.459
LVEF 35-55%	0.24 (0.10-0.60) P=0.002	0.17 (0.05-0.63) P=0.008	0.25 (0.10-0.62) P=0.003	0.32 (0.12-0.87) P=0.026
LVEF >55%	0.37 (0.16-0.88) P=0.025	0.39 (0.13-1.18) P=0.096	0.39 (0.16-0.92) P=0.031	0.83 (0.31-2.20) P=0.710
**Patients without CAD**				
LVEF ≤ 35%	0.48 (0.14-1.66) P=0.245	0.24 (0.05-1.09) P=0.064	0.50 (0.14-1.71) P=0.266	0.88 (0.21-3.72) P=0.856
LVEF 35-55%	0.19 (0.06-0.62) P=0.006	0.21 (0.05-0.94) P=0.042	0.20 (0.06-0.65) P=0.008	0.25 (0.07-0.92) P=0.037
LVEF >55%	0.30 (0.10-0.87) P=0.027	0.33 (0.11-1.03) P=0.056	0.32 (0.11-0.92) P=0.034	0.57 (0.17-1.94) P=0.367
**Patients without aortic disease**				
LVEF ≤ 35%	0.58 (0.20-1.69) P=0.317	0.54 (0.16-1.85) P=0.329	0.65 (0.22-1.88) P=0.425	1.15 (0.34-3.93) P=0.821
LVEF 35-55%	0.24 (0.09-0.67) P=0.006	0.11 (0.03-0.51) P=0.005	0.25 (0.09-0.69) P=0.008	0.31 (0.10-0.92) P=0.035
LVEF >55%	0.38 (0.13-1.07) P=0.067	0.41 (0.11-1.55) P=0.189	0.39 (0.14-1.12) P=0.079	0.82 (0.25-2.67) P=0.739
**Patients without secondary MR**				
LVEF ≤ 35%	0.55 (0.16-1.93) P=0.351	0.36 (0.09-1.44) P=0.149	0.57 (0.16-2.00) P=0.384	0.53 (0.11-2.43) P=0.410
LVEF 35-55%	0.21 (0.07-0.58) P=0.003	0.11 (0.03-0.38) P<0.001	0.21 (0.08-0.60) P=0.003	0.25 (0.08-0.79) P=0.017
LVEF >55%	0.44 (0.18-1.04) P=0.062	0.45 (0.15-1.34) P=0.152	0.45 (0.19-1.08) P=0.075	0.91 (0.34-2.47) P=0.853
**Patients without other cardiac or aortic surgery**				

LVEF ≤ 35%	1.14 (0.27-4.81)<br>P=0.862	1.14 (0.24-5.47)<br>P=0.873	1.17 (0.28-4.95)<br>P= 0.83	3.36 (0.66-17.19)<br>P=0.146
LVEF 35-55%	0.17 (0.04-0.71)<br>P=0.015	0.04 (0.01-0.24)<br>P=0.001	0.18 (0.04-0.73)<br>P=0.016	0.22 (0.05-0.96)<br>P=0.043
LVEF >55%	0.36 (0.11-1.17)<br>P=0.09	2.00 (0.37-10.91)<br>P=0.423	0.37 (0.12-1.21)<br>P=0.101	1.08 (0.29-4.01)<br>P=0.910

Abbreviations: CAD= coronary artery disease; CI= confidence interval; HHF= hospitalization for heart failure; HR= hazard ratio; IPTW= inverse probability of treatment weighting; LVEF= left ventricular ejection fraction; MR= mitral regurgitation

* IPTW Cox regression models based on the entire cohort, without any exclusion from the study population (n=1211).

† Cox proportional-hazard models based on the entire cohort, without any exclusion from the study population (n=1211), where AVI was treated as a time-dependent covariate.

Supplementary Table 7: Assessment of the impact of treatment strategies on the composite of death or HHF according to the LVEF ranges and reasons of admission

**Aortic valve intervention versus medical treatment**	**Univariate analysis of death or HHF** HR (95% CI); P value	**Multivariate analysis of death or HHF** HR (95% CI); P value
**Patients admitted for VHD related reasons ***		
LVEF ≤ 35%	0.25 (0.03-2.39) P=0.227	0.27 (0.03-2.81) P=0.272
LVEF 35-55%	0.16 (0.03-0.82) P=0.028	0.16 (0.03-0.85) P=0.031
LVEF >55%	0.60 (0.17-2.09) P=0.421	0.60 (0.15-2.46) P=0.481
**Patients admitted for non-VHD related reasons †**		
LVEF ≤ 35%	1.19 (0.35-4.06) P=0.781	1.06 (0.27-4.14) P=0.933
LVEF 35-55%	0.39 (0.12-1.24) P=0.110	0.22 (0.06-0.82) P=0.024
LVEF >55%	1.24 (0.23-6.65) P=0.804	1.28 (0.19-8.70) P=0.804

Abbreviations: CI= confidence interval; HHF= hospitalization for heart failure; HR= hazard ratio; LVEF= left ventricular ejection fraction; VHD= valvular heart disease

* indicating hospitalization for the diagnosis and treatment of valvular heart disease

† indicating hospitalization for cardiovascular diseases other than valvular heart disease, as well as non-cardiovascular disease

IV. Supplementary Figure

Figure Legend

Supplementary Figure 1: Hospitals participating in the China-VHD study and their geographical distribution.

Supplementary Figure 2: Flow chart of the study population.

Supplementary Figure 3: Variable selection for predicting death or HHF under medical treatment performed by the LASSO-penalized Cox regression and the coefficients of the selected variables. (A) The plot showing the deviance values of the LASSO model as a function of the tuning parameter λ. Tuning parameter λ selection in the LASSO model used 10-fold cross-validation via minimum criteria. The optimal λ is the value that minimizes the deviance curve (dashed line). (B) Trace plot showing non-zero model coefficients as a function of the tuning parameter λ. As the λ increases, LASSO sets various coefficients to zero, thus removing them from the model. (C) When λ corresponds to the minimum-deviance (dashed line), 12 variables are selected and their coefficients are listed in the table. BMI: body mass index; CABG: coronary artery bypass grafting; EuroSCORE-II: European System for Cardiac Operative Risk Evaluation; LVEF: left ventricular ejection fraction; LVESD: left ventricular end-systolic diameter; NYHA: New York Heart Association;

Supplementary Figure 4: Relative importance of the predictors of death or HHF under medical treatment selected by the LASSO-penalized Cox regression. The relative importance of predictors was determined by their computed proportional log-likelihood ratio χ^2 statistics. The models included variables selected by the LASSO model (**Supplementary Figure 3**), where LVEF was a continuous variable (model 1) or a categorical variable dichotomized by 55% (model 2). Abbreviations as in Supplementary figure 3.

Supplementary Figure 5: Variable selection for predicting death or HHF under aortic valve intervention treatment performed by the LASSO-penalized Cox regression and the coefficients of the selected variables. (A) The plot showing the deviance values of the LASSO model as a function of the tuning parameter λ. Tuning parameter λ selection in the LASSO model used 10-fold cross-validation via minimum criteria. The optimal λ is the value that minimizes the deviance curve (dashed line). (B) Trace plot showing non-zero model coefficients as a function of the tuning parameter λ. As the λ increases, LASSO sets various coefficients to zero, thus removing them from the model. (C) When λ corresponds to the minimum-deviance (dashed line), 5 variables are selected and their coefficients are listed in the table. Abbreviations as in Supplementary figure 3.

Supplementary Figure 6: Relative importance of the predictors of death or HHF under aortic valve intervention treatment selected by the LASSO-penalized Cox regression. The relative importance of predictors was determined by their computed proportional log-likelihood ratio χ^2 statistics. The models included variables selected by the LASSO model (**Supplementary Figure 5**), where LVEF was a continuous variable. Abbreviations as in Supplementary figure 3.

Supplementary Figure 7: Determination of the best LVEF cutoff value for predicting death or HHF under medical treatment using the maximally selected rank statistics method and the relative risk with the selected cutoff as reference. (A) The composite outcome of death or HHF under medical treatment was assessed in the whole cohort, with censoring at the time of aortic valve intervention if performed. The maximally selected rank statistics method is an outcome-oriented method providing a cutoff value that corresponds to the most significant relation with the outcome, with the largest standardized log-rank statistics over all possible cutoff points. The plot illustrates the distribution pattern of patients and the

standard log-rank statistics of all possible LVEF cutoff. The value at which standard log-rank statistics reached maximum was selected as the best cutoff (Dash line). (B) Penalized spline showing the relative risk of death or HHF after AVI versus under medical treatment according to LVEF, with the selected cutoff (LVEF=55%) as reference

Supplementary Figure 8: Decision curve analysis of the prediction models with and without baseline LVEF for the composite of death or HHF under medical treatment. The decision curve analysis curves graphically show the clinical usefulness of each model based on a continuum of potential thresholds for the primary outcome (x axis) and the net benefit of using the models to risk-stratify patients (y axis) relative to assuming that no patient will die or be hospitalized for heart failure. Thus, the higher curve at a given threshold probability is optimal. The base model included age, body mass index, atrial fibrillation, prior myocardial infarction, prior coronary artery bypass grafting, chronic kidney disease, New York Heart Association class III/IV, hemoglobin, left ventricular end-systolic diameter >50 mm, pulmonary hypertension, and EuroSCORE-II. Abbreviations as in Supplementary figure 3.

Supplementary Figure 9: Association between baseline LVEF and relative hazard of 2-year death or HHF in age- and sex-matched population without left-sided valvular heart disease. Penalized spline curves demonstrate the shape of the association in age- and sex-matched population without left-sided VHD using 1:1 propensity score matching method (n=1211), with 95% confidence interval. The grey area underneath the curve indicates the density of the population. The horizontal line at HR=1 represents the mean risk of the cohort. Abbreviations as in Supplementary figure 3.

Supplementary Figure 10: Determination of the best LVEF cutoff value for predicting death or HHF in age- and sex-matched population without left-sided valvular heart disease using the maximally selected rank statistics method, and the relative risk with the

selected cut-point as reference, and Kaplan-Meier curves of event-free survival. (A) The maximally selected rank statistics method is an outcome-oriented method providing a cutoff value that corresponds to the most significant relation with the outcome, with the largest standardized log-rank statistics over all possible cutoff points. The plot illustrates the distribution pattern of patients and the standard log-rank statistics of all possible LVEF cutoff. The value at which standard log-rank statistics reached maximum was selected as the best cutoff (Dash line). (B) Penalized spline showing the relative risk of death or HHF after AVI versus under medical treatment according to LVEF, with the selected cutoff (LVEF=55%) as reference. Abbreviations as in Supplementary figure 3.

Supplementary Figure 11: Graphical inspection of scaled Schoenfeld residuals to test the proportional hazard assumption. The graphs show the scaled Schoenfeld residuals against the transformed follow-up time. The solid line is a smoothing spline fit to the plot, with the dashed lines representing a 2-standard-error band around the fit.

Supplementary Figure 12. Absolute standardized mean differences across covariates before and after the inverse probability of treatment weighted adjustment. The plot illustrates the balance of covariates between the patients under medical treatment and aortic valve intervention before (Green) and after (Red) the IPTW adjustment. Absolute standardized mean differences <20% (Dash line) indicates good comparability between the two groups.

Supplementary Figure 1: Hospitals participating in the China-VHD study and their geographical distribution.

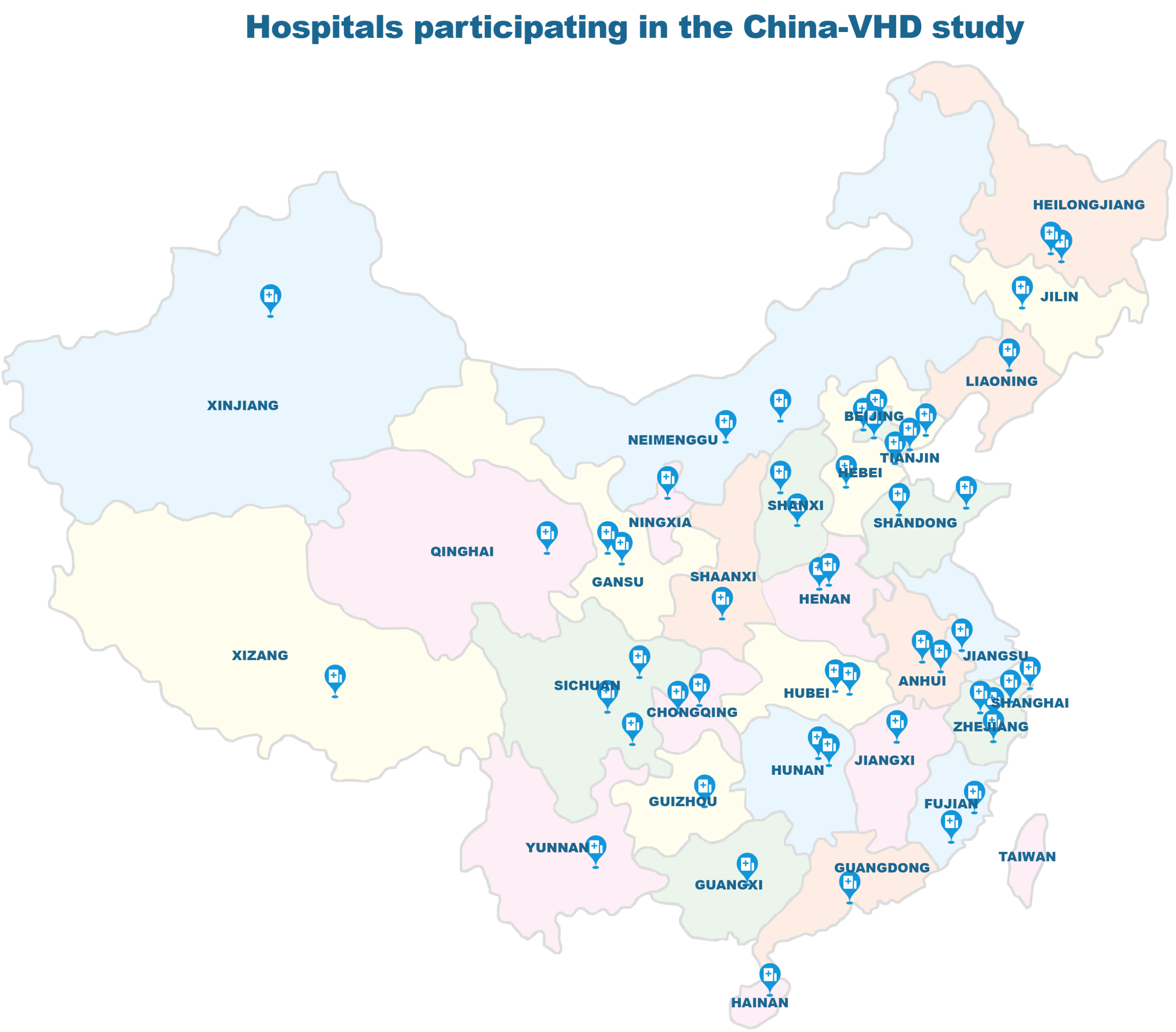

Hospital	Province/Municipality	Local PI
Fuwai Hospital	Beijing	Yongjian Wu, Haiyan Xu
Beijing Anzhen Hospital	Beijing	Yujie Zhou, Haibo Zhang
Chinese PLA General Hospital	Beijing	Changfu Liu
Zhongshan Hospital affiliated to Fudan University	Shanghai	Daxin Zhou
Shanghai Changhai Hospital	Shanghai	Xianxian Zhao
Tianjin Medical University General Hospital	Tianjin	Qing Yang

Hospital	Province/Municipality	Local PI
Tianjin Chest Hospital	Tianjin	Hongliang Cong
Southwest Hospital	Chongqing	Zhihui Zhang
Yijishan Hospital affiliated to Wannan Medical School	Anhui	Yongsheng Ke
Union Hospital Affiliated to Fujian Medical University	Fujian	Lianglong Chen
Quanzhou First Hospital	Fujian	Rong Lin
The First Hospital Affiliated to Lanzhou University	Gansu	Zheng Zhang, Bing Song
Gansu Provincial Hospital	Gansu	Ping Xie
Guangdong Provincial Hospital	Guangdong	Jianfang Luo
The First Hospital affiliated to Guangxi Medical University	Guangxi	Hong Wen
Guizhou Provincial Hospital	Guizhou	Qiang Wu
Hebei Provincial Hospital	Hebei	Xiaoyong Qi
Tangshan Workers' Hospital	Hebei	Zheng Ji
Henan Provincial Hospital	Henan	Chuanyu Gao
The First Affiliated Hospital of Zhengzhou University	Henan	Ling Li
The First Hospital affiliated to Haerbin Medical University	Heilongjiang	Yue Li
The Second Hospital affiliated to Haerbin Medical University	Heilongjiang	Bo Yu
Wuhan Asia Heart Hospital	Hubei	Xi Su
The Second Xiangya Hospital of Central South University	Hunan	Xinqun Hu
The Second Hospital affiliated to Jilin University	Jilin	Bin Liu
Nanjing First Hospital	Jiangsu	Shaoliang Chen
The First Hospital affiliated to Nanchang University	Jiangxi	Zeqi Zheng
Inner Mongolia People's Hospital	Inner Mongolia	Xingsheng Zhao
The First Hospital affiliated to Baotou Medical College	Inner Mongolia	Hanjun Pei
General Hospital affiliated to Ningxia Medical University	Ningxia	Hui Huang
The Affiliated Hospital of Qinghai University	Qinghai	Ming Ren
Qingdao Fuwai Hospital	Shandong	Xianyan Jiang
Qilu Hospital affiliated to Shandong University	Shandong	Mei Zhang
The First Hospital affiliated to Shanxi Medical University	Shanxi	Qinghua Han
Shanxi Cardiovascular Hospital	Shanxi	Jian An

Hospital	Province/Municipality	Local PI
Xijing Hospital	Shaanxi	Ling Tao, Fei Li
West China Hospital affiliated to Sichuan University	Sichuan	Yingqiang Guo
The Affiliated Hospital of Southwest Medical University	Sichuan	Zhongcai Fan
Nanchong Central Hospital	Sichuan	Haoyu Wang
The People's Hospital of the Tibet Autonomous Region	Tibet	Luobu Gesang
The First Hospital affiliated to Xinjiang Medical University	Xinjiang	Xiang Ma
The First Hospital affiliated to Kunming Medical University	Yunnan	Tao Guo
The Second Hospital affiliated to Zhejiang University	Zhejiang	Jianan Wang
Shaoyifu Hospital affiliated to Zhejiang University	Zhejiang	Guosheng Fu
Hainan provincial Hospital	Hainan	Wang Liao
The First Hospital affiliated to Jinzhou Medical University	Liaoning	Guizhou Tao
**A total of 46 hospitals from 27 provinces and 4 municipalities participated in the study.**		
**Principle investigators: Yongjian Wu and Haiyan Xu**		

Supplementary Figure 2: Flow chart of the study population

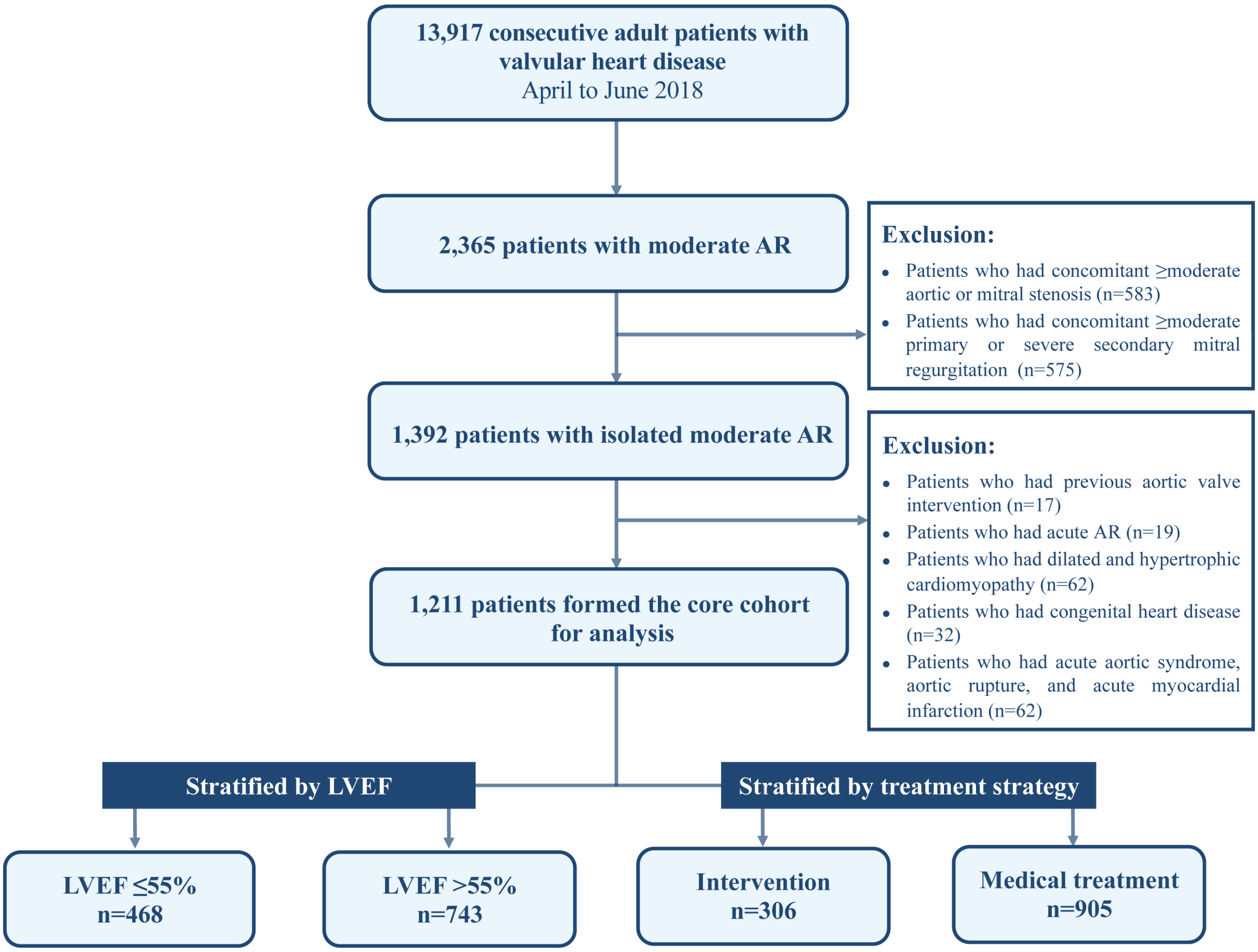

Supplementary Figure 3: Variable selection for predicting death or HHF under medical treatment performed by LASSO-penalized Cox regression and the coefficients of the selected variables. (Figure Legend: Supplementary material P23)

A.

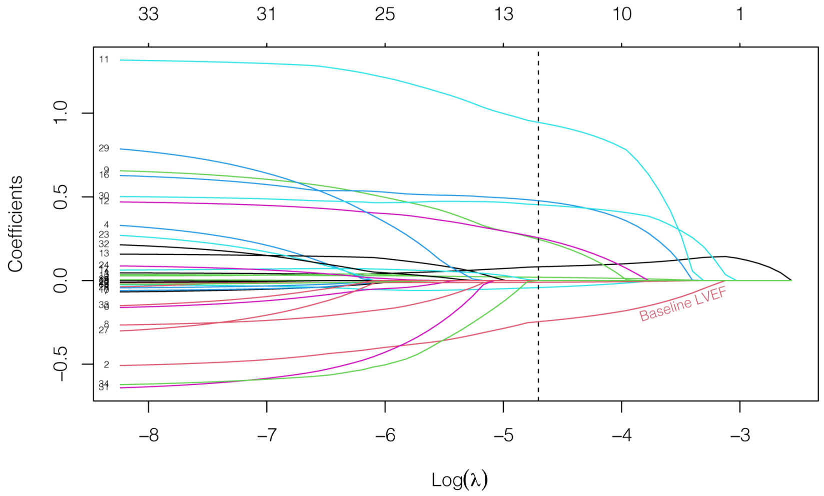

B.

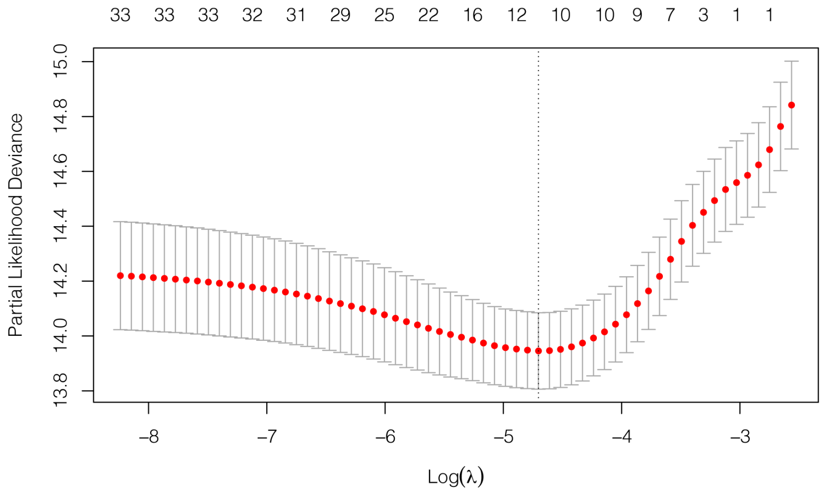

C.

**Variables**	**HR (95%CI)**	**P value**
LVEF (per 10% increase)	0.65 (0.53-0.79)	0.008
Age	1.03 (1.01-1.05)	0.003
BMI	0.93 (0.88-0.98)	0.010
Myocardial infarction	1.55 (0.87-2.74)	0.135
Prior CABG	3.28 (1.45-7.39)	0.004
Atrial fibrillation	1.51 (1.01-2.26)	0.047
Chronic kidney disease	1.69 (0.98-2.93)	0.061
NYHA functional class III/IV	1.13 (0.74-1.80)	0.569
Hemoglobin	0.99 (0.98-0.99)	0.008
LVESD >50 mm	0.52 (0.28-0.97)	0.039
Pulmonary hypertension	1.68 (1.12-2.53)	0.012
EuroSCORE-II	1.04 (0.95-1.13)	0.434
**C-statistics = 0.78**		

Supplementary Figure 4: Relative importance of predictors of death or HHF under medical treatment selected by LASSO-penalized Cox regression. (Figure Legend: Supplementary material P23)

Model 1: LVEF as continuous variable

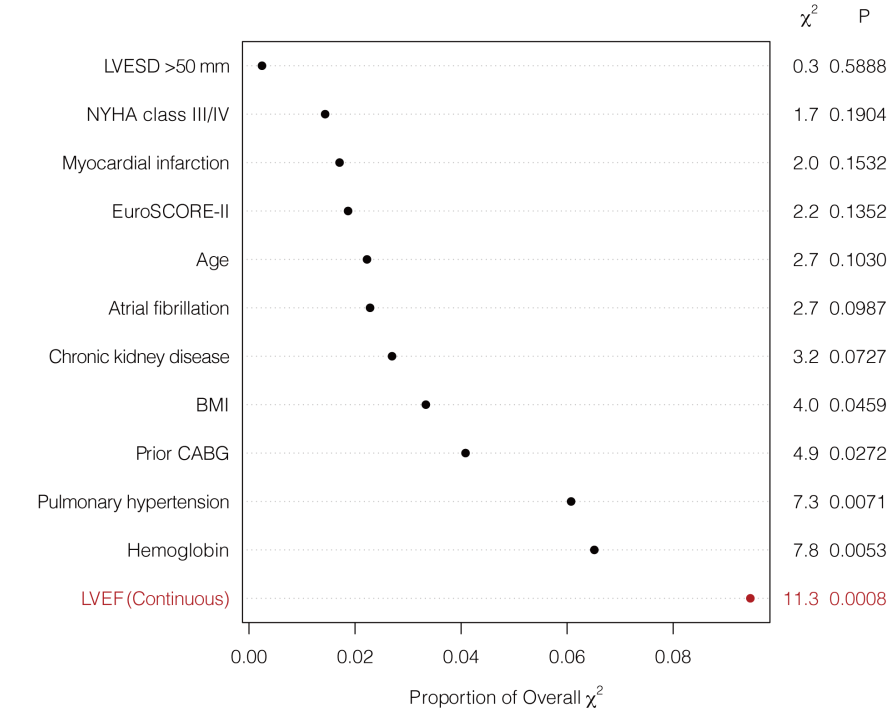

Model 2: LVEF dichotomized by 55%

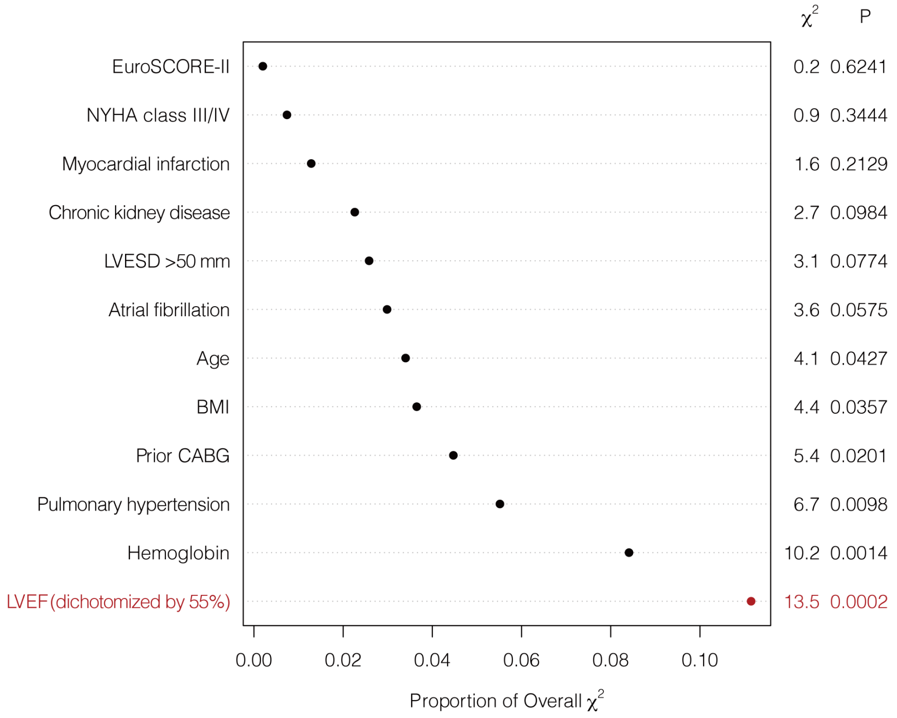

Supplementary Figure 5: Variable selection for predicting death or HHF under aortic valve intervention treatment performed by LASSO-penalized Cox regression and the coefficients of the selected variables. (Figure Legend: Supplementary material P24)

A.

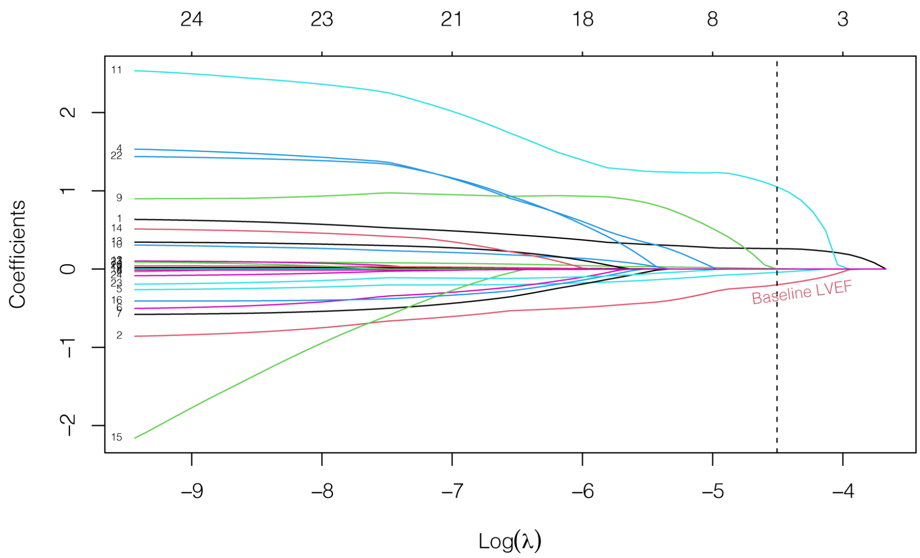

B.

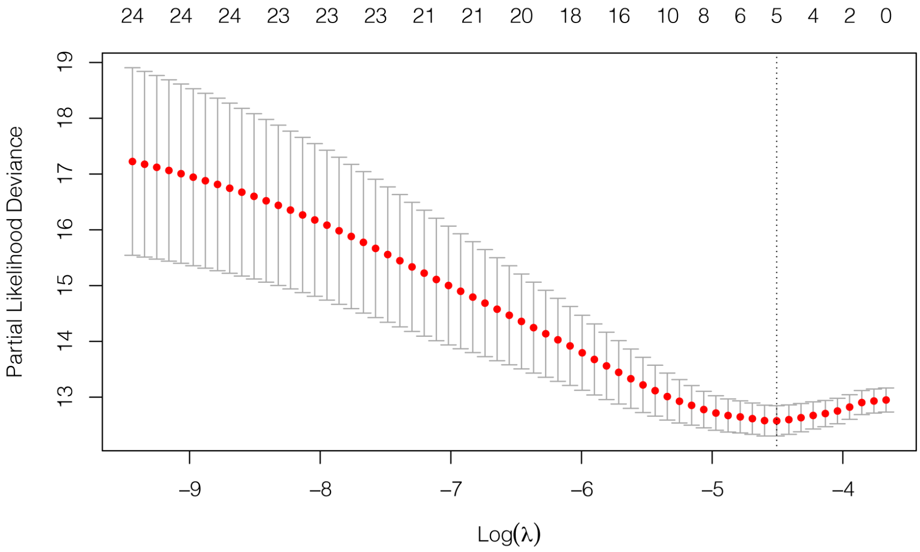

C.

**Variables**	**HR (95%CI)**	**P value**
LVEF (per 10% increase)	0.65 (0.43-0.97)	0.036
Age	1.05 (1.00-1.10)	0.076
BMI	0.86 (0.73-1.03)	0.100
Prior CABG	4.28 (0.49-37.68)	0.190
EuroSCORE-II	1.25 (0.87-1.80)	0.225
**C-statistics = 0.75**		

Supplementary Figure 6: Relative importance of predictors of 2-year death or HHF under aortic valve intervention treatment selected by LASSO-penalized Cox regression.

(Figure Legend: Supplementary material P24)

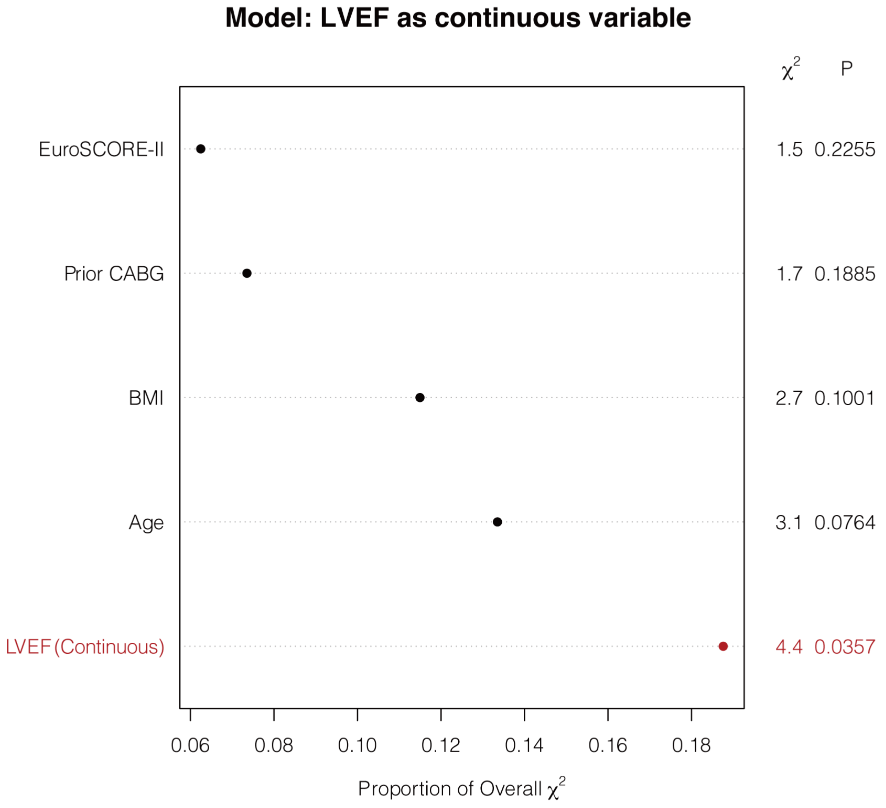

Supplementary Figure 7: Determination of the best LVEF cutoff value for predicting death or HHF under medical treatment using the maximally selected rank statistics method and the relative risk with the selected cut-point as reference. (Figure Legend: Supplementary material P24-25)

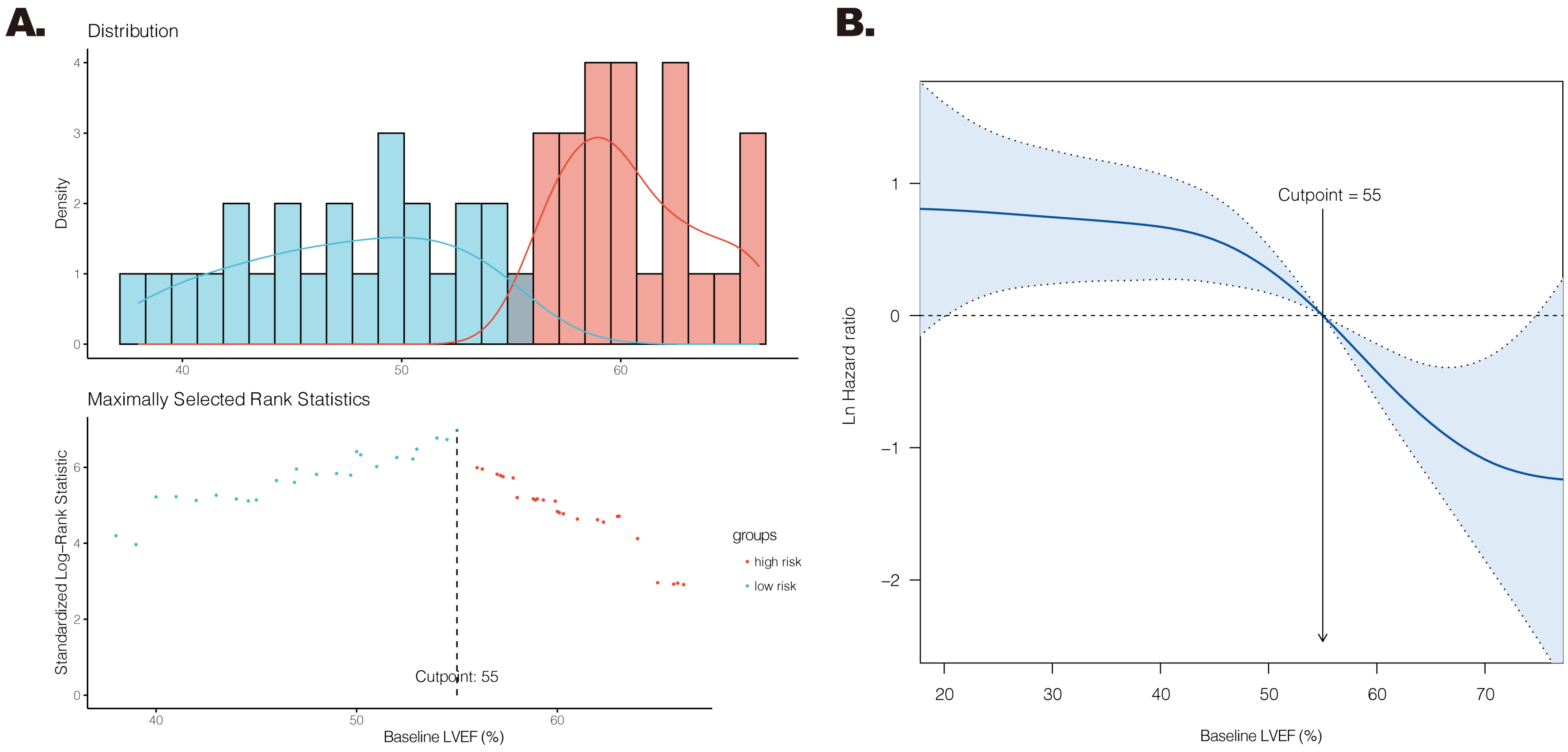

Supplementary Figure 8: Decision curve analysis of prediction models with and without baseline LVEF for the composite outcome of death or HHF under medical treatment.

(Figure Legend: Supplementary material P25)

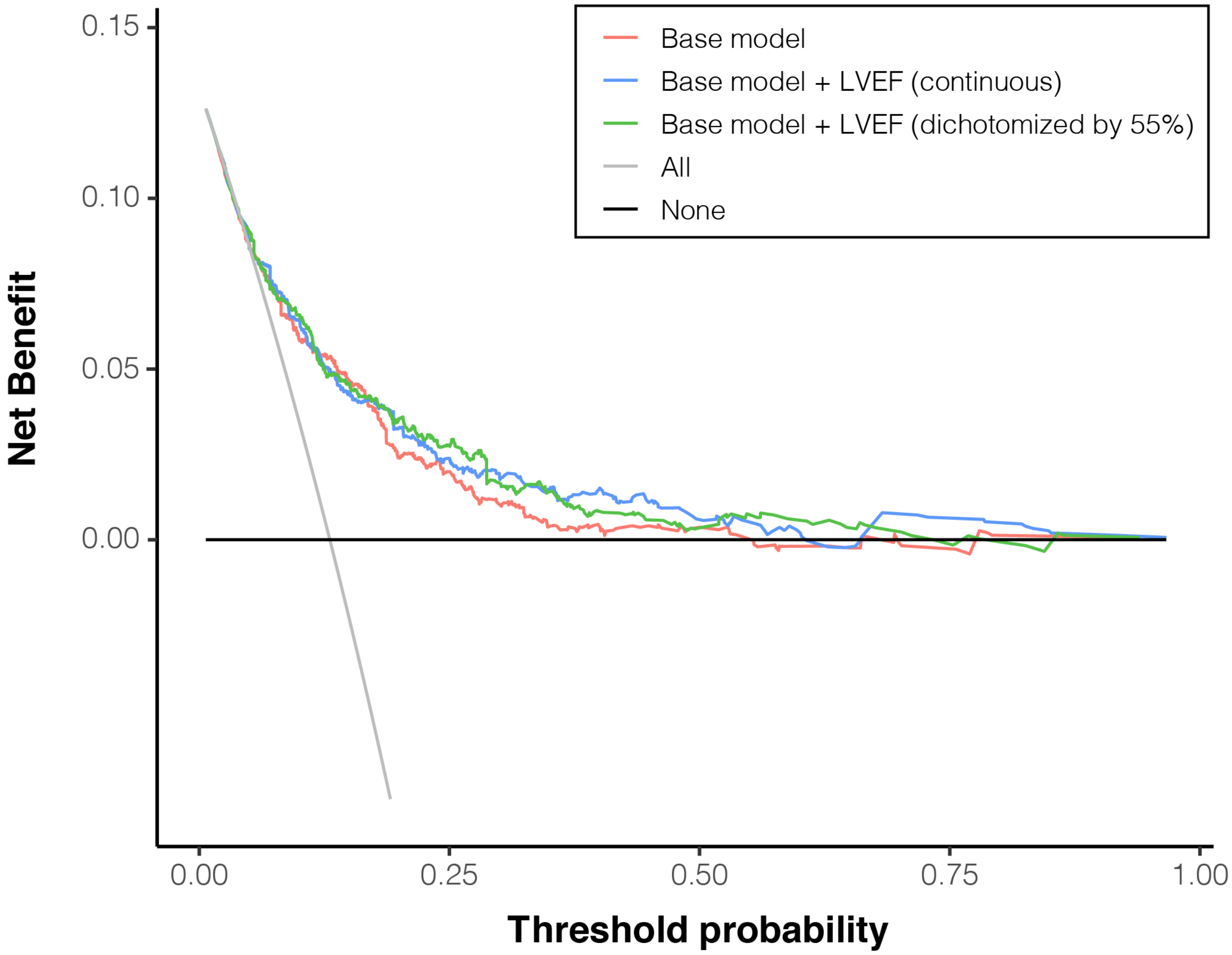

Supplementary Figure 9: Association between baseline LVEF and relative hazard of 2-year death or HHF in age- and sex-matched population without left-sided valvular heart disease. (Figure Legend: Supplementary material P25)

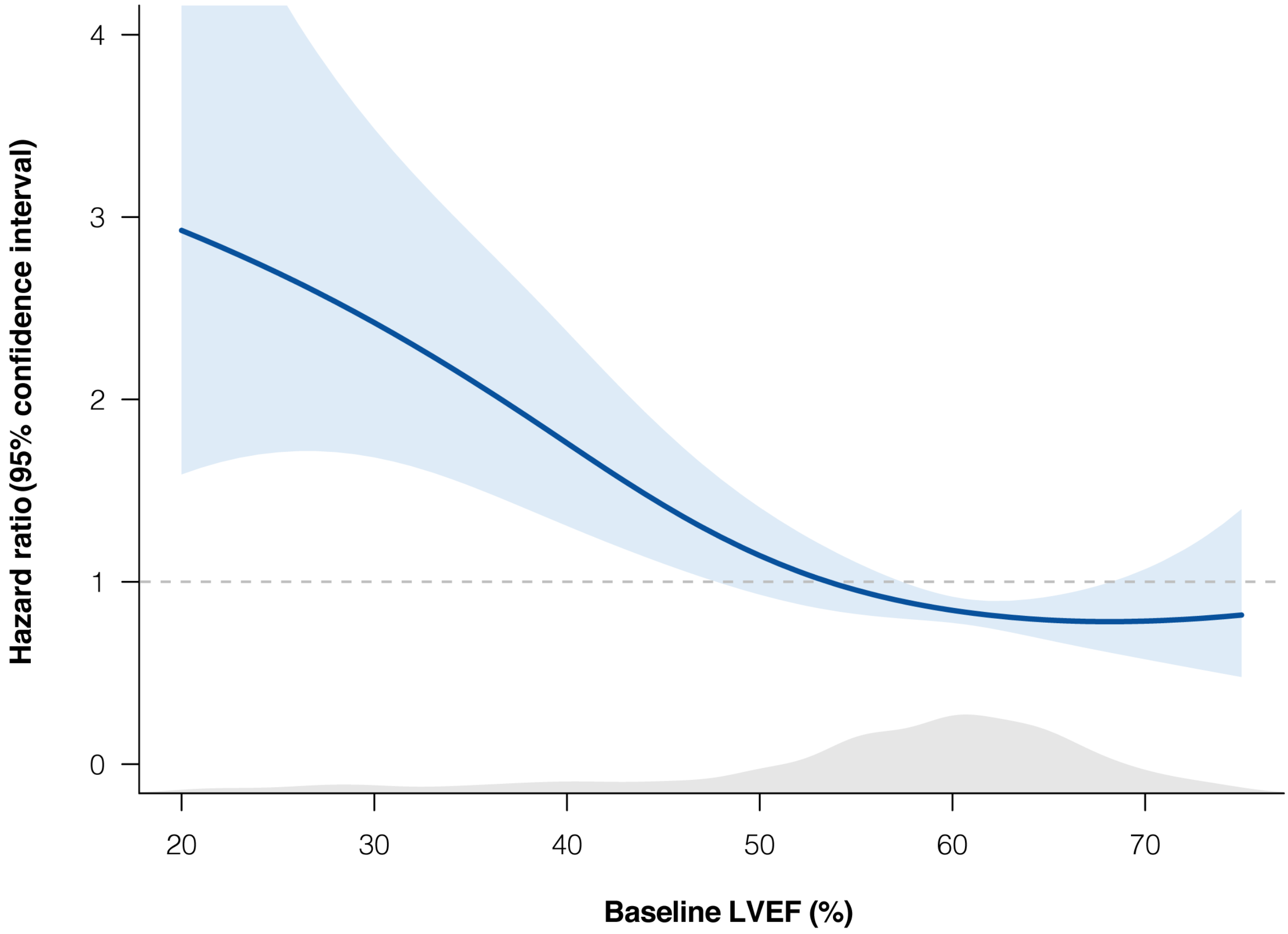

Supplementary Figure 10: Determination of the best LVEF cutoff value for predicting death or HHF in age- and sex-matched population without left-sided valvular heart disease using the maximally selected rank statistics method, and the relative risk with the selected cut-point as reference, and Kaplan-Meier curves of event-free survival. (Figure Legend: Supplementary material P25-26)

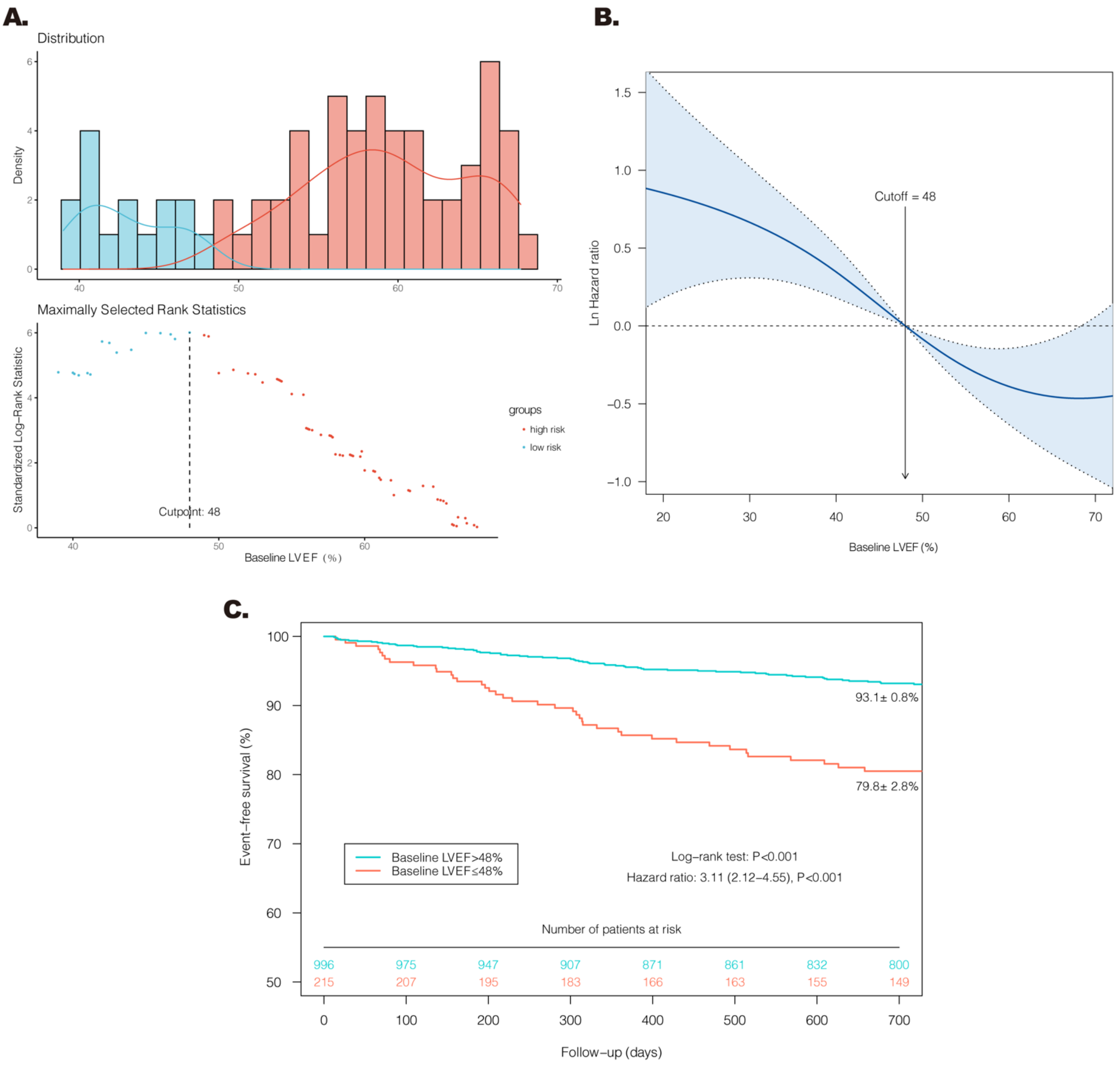

Supplementary Figure 11: Graphical inspection of scaled Schoenfeld residuals to test the proportional hazard assumption. (Figure Legend: Supplementary material P26)

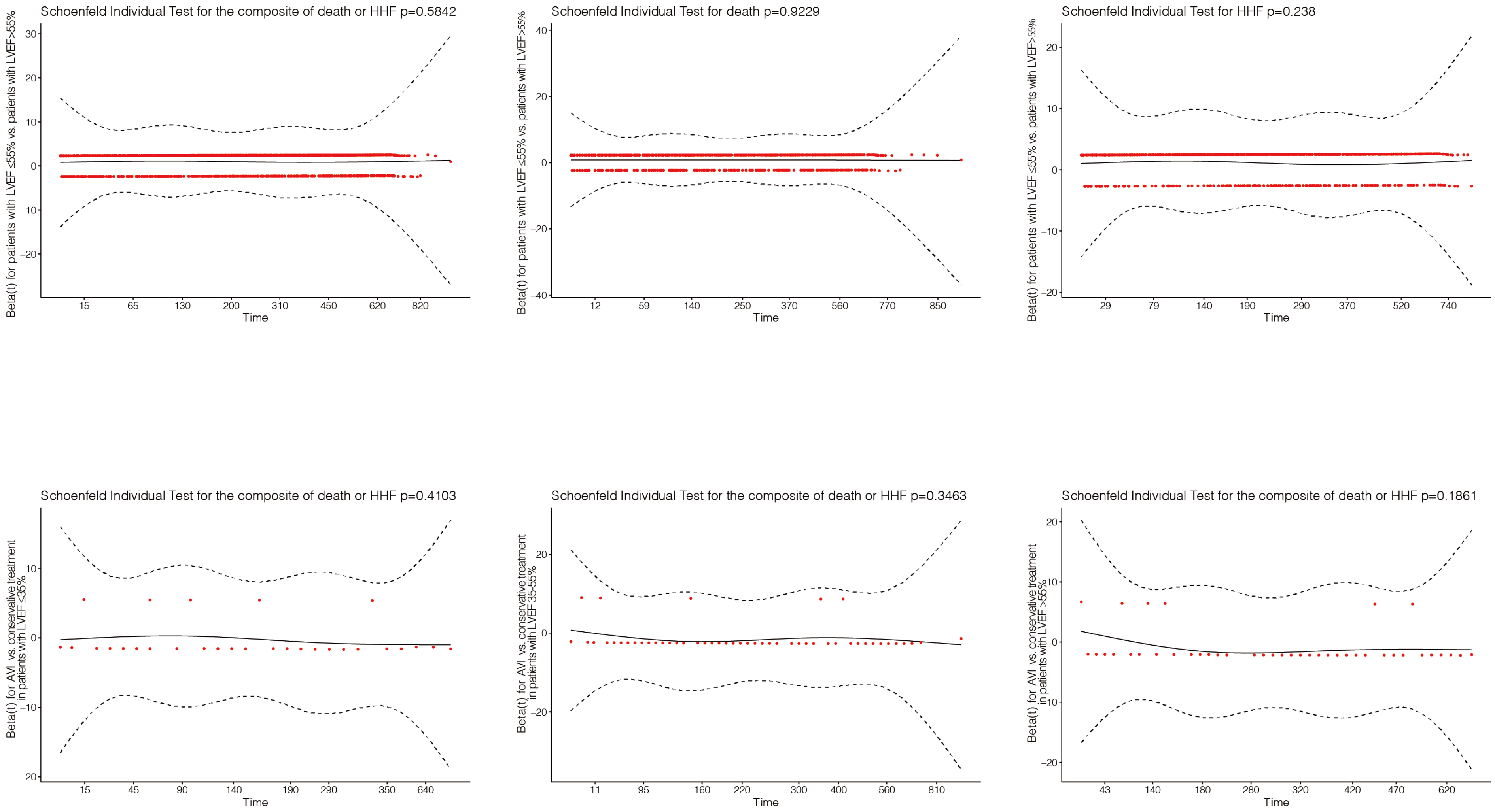

Supplementary Figure 12. Absolute standardized mean differences across covariates before and after the inverse probability of treatment weighted adjustment. (Figure Legend: Supplementary material P26)

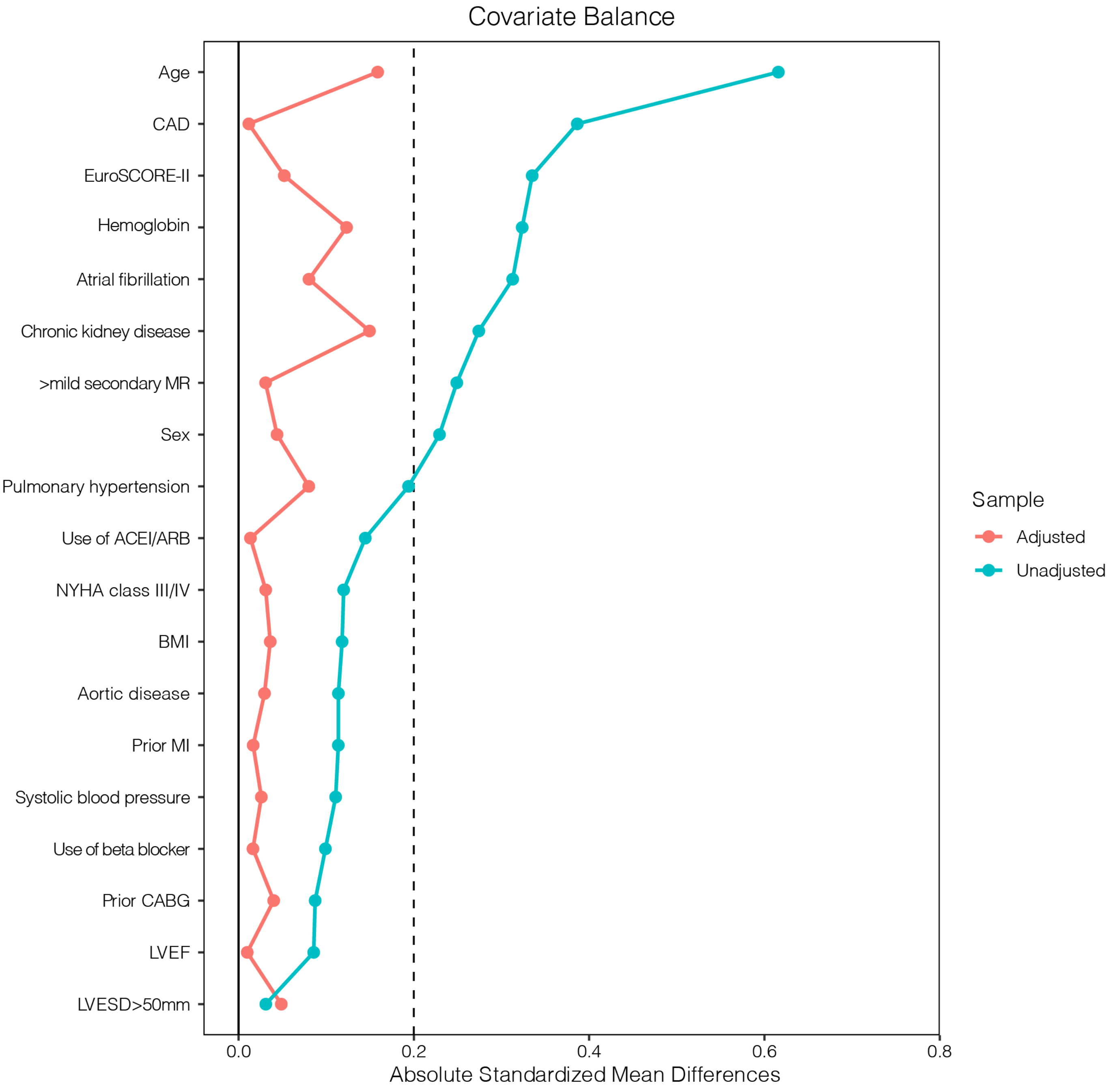

Supplement: Supplementary file 1 [file Data_Sheet_1.pdf]
